# Altered striatal long-term potentiation in the eIF4E-TG mouse model of autism spectrum disorder

**DOI:** 10.1016/j.isci.2026.117278

**Published:** 2026-09-03

**Authors:** Alina Aaltonen, Jone Razquin, Daniel R. Garton, Julia Oyrer, Chiara Criscuolo, Ori J. Lieberman, Eric Klann, Anders Borgkvist, Emanuela Santini

**Affiliations:** 1 Department of Neuroscience, Karolinska Institutet, Stockholm, Sweden; 2 Department of Pharmacology, Columbia University College of Physicians and Surgeons, New York, NY 10032, USA; 3 Department of Neurology, University of California, San Francisco, CA, USA; 4 Center for Neural Science, New York University, New York, NY, USA

**Keywords:** autism spectrum disorder, eIF4E, striatum, synaptic plasticity, long-term potentiation, spiny projection neurons, dendritic spines, NMDA receptors, dopamine, translational control

## Abstract

Autism spectrum disorder (ASD) is associated with deficits in synaptic plasticity across multiple brain regions. While striatal dysfunction is observed in several ASD mouse models, the effects of ASD-associated genes on striatal plasticity remain poorly understood. We previously showed that overexpression of the Simons Foundation Autism Research Initiative (SFARI) ASD risk gene eukaryotic initiation factor 4E (*eIF4E*) in transgenic (eIF4E-TG) mice produces ASD-like behaviors and impairs dorsal striatal dopamine release. Here, we examined whether eIF4E overexpression alters striatal synaptic transmission and plasticity. eIF4E-TG mice exhibited increased dendritic spine density, elevated AMPA- and NMDA receptor-mediated miniature excitatory postsynaptic current (mEPSC) frequency, and reduced AMPA mEPSC amplitude. In addition, spiny projection neurons (SPNs) showed enhanced induction and magnitude of NMDA receptor-dependent long-term potentiation (LTP) that was not prevented by D1 or D2 receptor antagonism. Finally, depolarization-evoked somatic and dendritic Ca^2+^ signals were altered in eIF4E-TG SPNs. Together, these findings indicate that eIF4E overexpression biases striatal neurons toward NMDA receptor-dependent LTP.

## Highlights

• eIF4E overexpression increases dendritic spine density in striatal SPNs
• Excitatory and inhibitory synaptic transmission is altered in eIF4E-TG mice
• eIF4E overexpression enhances NMDA receptor-dependent striatal LTP
• eIF4E overexpression alters dendrite-to-soma Ca^2+^ signaling in SPNs

## Graphical abstract

**Figure:**
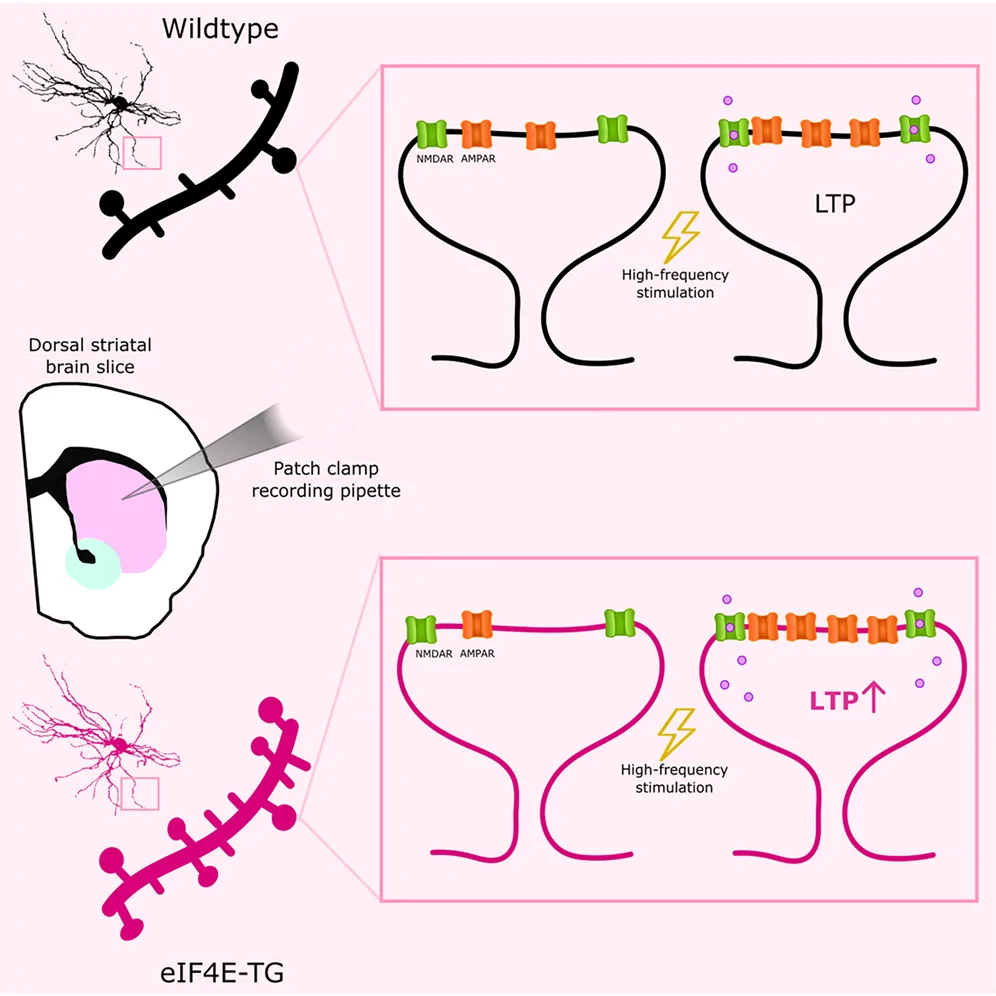

## Teaser

Disease; Neurogenetics; Neuroscience

## Introduction

Synaptic plasticity occurs throughout the brain and underlies development and learning.^1^^,^^2^^,^^3^^,^^4^ The expression of synaptic plasticity in forms of long-term potentiation (LTP) and depression (LTD) is predominantly observed at the level of dendritic spines and synapses.^5^^,^^6^^,^^7^ LTP and LTD are regulated by many effectors and modulators, such as glutamatergic receptors^8^^,^^9^ and neuromodulators.^10^^,^^11^^,^^12^^,^^13^ In addition, the importance of protein synthesis in various aspects of synaptic plasticity has been established.^14^^,^^15^^,^^16^^,^^17^^,^^18^^,^^19^^,^^20^ Neuronal activity-dependent protein synthesis is thought to influence not only the magnitude of synaptic change but also the thresholds and persistence of plasticity, thereby shaping the rules by which synapses undergo long-term modification across circuits. These regulatory mechanisms operate across brain regions, but their functional consequences may differ depending on circuit architecture and neuromodulatory context.

Dysregulation of protein synthesis via mTORC1 signaling is implicated in autism spectrum disorder (ASD), both in idiopathic autism^21^^,^^22^^,^^23^ and associated genetic syndromes.^24^^,^^25^^,^^26^^,^^27^^,^^28^^,^^29^^,^^30^ Mutations disrupting the mTORC1 pathway correlate with altered dendritic spine density and morphology across various brain regions,^31^^,^^32^^,^^33^^,^^34^^,^^35^^,^^36^^,^^37^^,^^38^^,^^39^ as well as excitation-inhibition imbalance^40^^,^^41^ and numerous aberrant forms of synaptic plasticity.^25^^,^^36^^,^^42^^,^^43^^,^^44^^,^^45^^,^^46^^,^^47^

One circuit in which neuromodulatory context critically shapes plasticity is the striatum. Striatal dendritic spines, which harbor corticostriatal synapses, exhibit various forms of plasticity, including NMDA receptor (NMDAR)-dependent LTP^48^^,^^49^ and endocannabinoid (eCB)-dependent LTD.^49^^,^^50^^,^^51^^,^^52^ Many forms of striatal LTP/LTD are modulated by dopamine and show dopamine dependence that varies with induction protocol and circuit state.^12^^,^^48^^,^^52^^,^^53^^,^^54^ Studies in ASD models reveal disrupted corticostriatal functional connectivity,^55^^,^^56^^,^^57^^,^^58^^,^^59^ suggesting that the striatum is affected by ASD-like synaptic plasticity dysregulation. In addition, fragile X syndrome (FXS) model mice have alterations in striatal spiny projection neurons (SPNs) including dendritic spines, altered actin turnover and striatal LTD, which correlate with repetitive behaviors and behavioral inflexibility.^26^^,^^60^^,^^61^ TSC1 knockout also results in impaired eCB-LTD.^59^ While there is a growing literature on striatal LTD in ASD models, it remains unclear whether LTP is similarly altered and whether such changes contribute to ASD-relevant circuit dysfunction. In particular, dysregulated striatal LTP is observed in mouse models of other striatal disorders, such as addiction disorders and Parkinson’s disease.^62^^,^^63^ Given the central role of the striatum in action selection, habit formation, and behavioral flexibility, even subtle alterations in synaptic plasticity rules may have disproportionate consequences for circuit output and ASD-relevant behavioral phenotypes.

Eukaryotic initiation factor 4E (eIF4E) acts downstream of the mTORC1 pathway and is listed as an SFARI high-confidence ASD candidate gene,^64^ with rare variants implicated in autism.^65^ eIF4E-TG (transgenic) and 4E-BP2 (repressor) knockout mice display ASD-relevant behavioral and synaptic phenotypes across brain regions.^66^^,^^67^^,^^68^^,^^69^^,^^70^^,^^71^ We previously identified enhanced striatal LTD in the eIF4E-TG mice,^70^ and more recently a deficit in dorsal striatal dopamine release,^68^ supporting altered striatal circuit function in this model. In the present study, we combined whole-cell patch clamp electrophysiology, optogenetics, fast-scan cyclic voltammetry and two-photon microscopy to test whether eIF4E overexpression alters excitatory synaptic structure and function in SPNs and modifies rules for long-term plasticity. We report increased SPN spine density, miniature current (miniature excitatory postsynaptic current [mEPSC]) frequency and reduced AMPA receptor-mediated mEPSC amplitude. While optogenetic stimulation of sensorimotor corticostriatal inputs did not reveal a change in baseline synaptic connectivity, we observed enhanced NMDAR-dependent potentiation induced by afferent stimulation under disinhibited recording conditions, which was not prevented by D1- or D2-receptor antagonism at the concentrations tested. Together, these data define an altered striatal plasticity profile in eIF4E-TG mice and motivate future work to determine the circuit and neuromodulatory requirements of this potentiation.

## Results

### Increased dendritic spines in striatal SPNs of the eIF4E-TG mice

**Figure 1:**
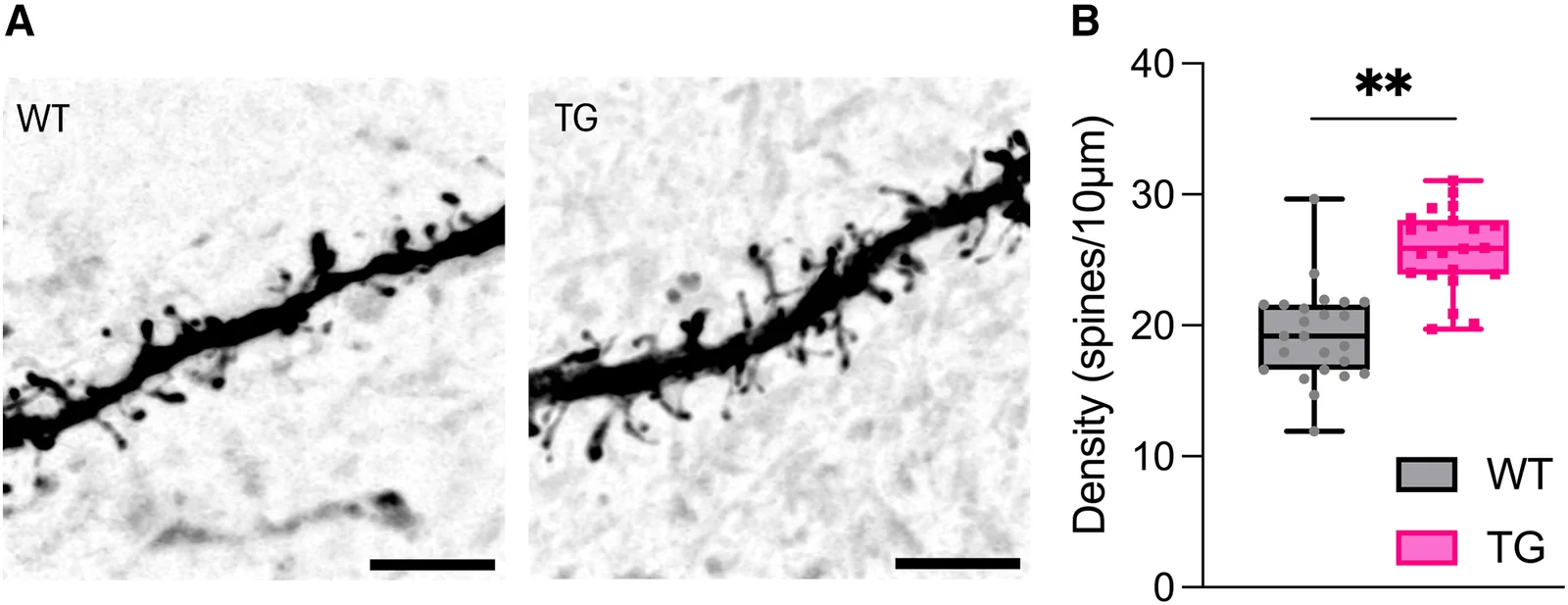
Dendritic spines of SPNs of WT and eIF4E-TG mice (A and B) Images were collected from 3 WT and 3 TG mice (neurons *n* = 24 WT and *n* = 22 TG). (A) Example Airyscan confocal images of SPN dendrites (scale bars, 4 μm). (B) SPN dendritic spine density in WT and eIF4E-TG mice (linear mixed-effects model with mouse as a random effect, ∗∗*p* = 0.0045). Data are presented as median ± quartiles within the boxplot, with whiskers indicating minimum and maximum values. Dots represent individual neurons. Significance is denoted as ∗∗*p* < 0.01. For full details of statistical analysis, including negative results, refer to Table S1.

Altered dendritic spine density and morphology are reported in ASD postmortem studies.^38^^,^^39^ Consistently, dendritic spine alterations are also reported across multiple ASD mouse models and brain regions.^26^^,^^31^^,^^32^^,^^33^^,^^34^^,^^35^^,^^36^^,^^37^^,^^72^ We have previously identified changes in dendritic spine density in other regions of the eIF4E-TG brain that correlated with altered synaptic function,^70^ while dendritic arborization of SPNs was unchanged.^69^ Because dendritic spine density in the striatum had not been assessed in this model, we quantified SPN spine density in the eIF4E-TG and wild-type (WT) mice. Individual SPNs were microinjected with 8% Lucifer Yellow CH fluorescent dye and distal dendritic segments (50 μm from the soma) were analyzed in 2 to 3-month-old male and female littermates. We observed a significant increase in spine density in SPNs of the eIF4E-TG mice (linear mixed-effects model with mouse as a random effect; Figures 1A and 1B and Table S1). This increase was observed across dendrites, neurons, and animals and was present in both D1-and D2-SPNs, with no significant genotype × cell-type interaction detected (Table S7). Additional analysis of dendritic spine morphology did not reveal significant genotype-dependent differences in the relative distribution of spine subtypes (Figure S1 and related Table S6).

### Altered spontaneous synaptic transmission in the eIF4E-TG SPNs

As we observed an increase in dendritic spine density in the SPNs of TG mice, and distal dendrites on SPNs are preferentially innervated by excitatory afferents,^73^^,^^74^ we hypothesized that excitatory synaptic transmission may be altered. We performed voltage-clamp recordings in whole-cell configuration of mEPSCs and miniature inhibitory PSCs (mIPSCs) in SPNs of 2 to 3-month-old D1-TdTomato/WT and D1-TdTomato/TG or D2-eGFP/WT and D2-eGFP/TG mice (see STAR Methods). As no genotype X cell-type interaction effects were detected for dendritic spine density or in mEPSC/mIPSCs parameters (Table S7), recordings from D1-and D2-SPNs were pooled for subsequent analyses. In addition, no genotype × sex interaction effects were detected for mEPSC/mIPSC parameters (Table S8), and recordings from male and female mice were therefore analyzed together.

**Figure 2:**
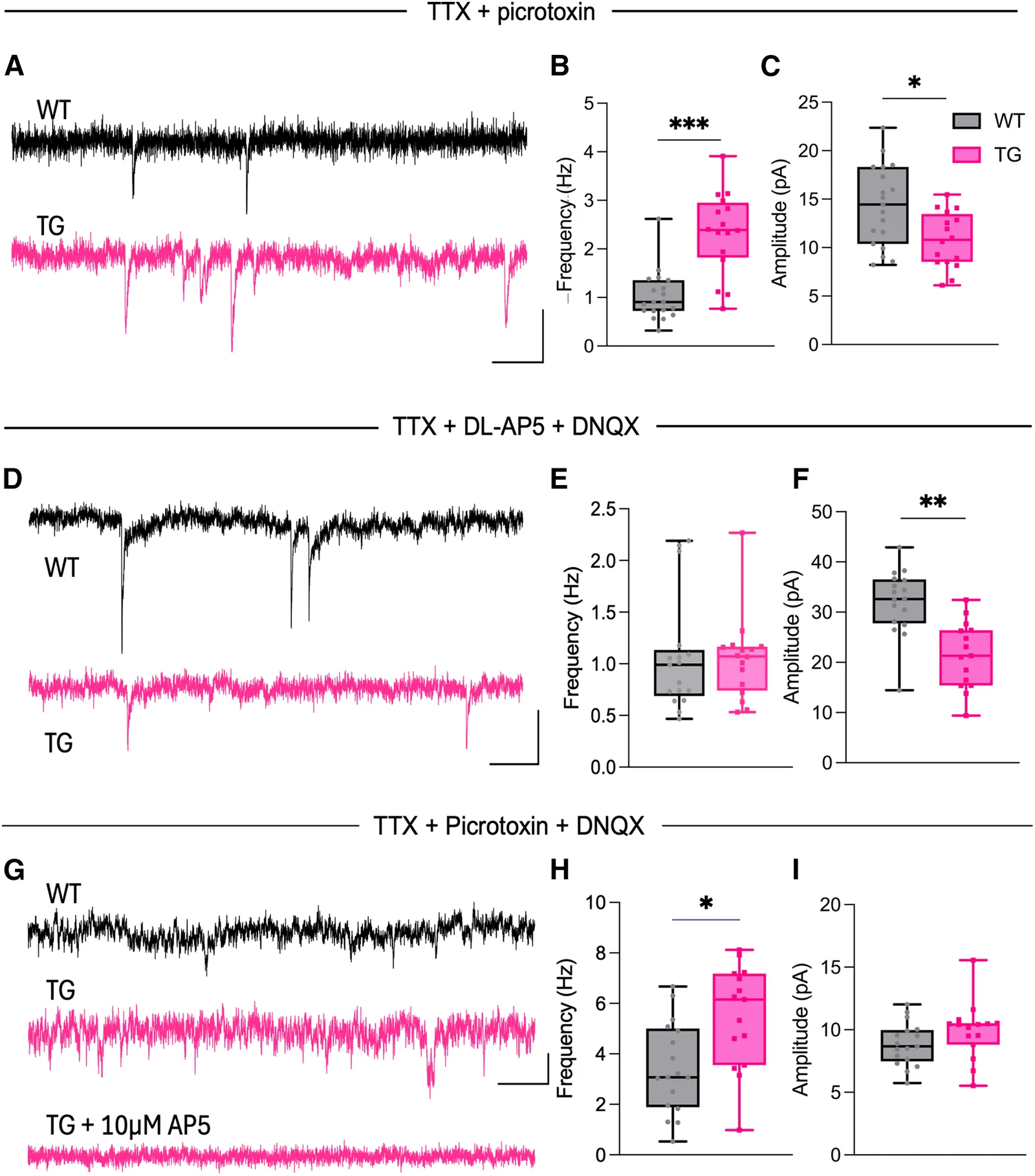
mEPSCs and mIPSCs from WT and eIF4E-TG SPNs (A–C) Recordings were collected from 7 WT and 5 TG mice (neurons *n* = 19 WT and *n* = 16 TG). (A) Representative mEPSC traces recorded with bath application of 1 μM TTX and 100 μM picrotoxin (scale bars: 20 pA, 200 ms). (B) mEPSC frequency in WT and eIF4E-TG mice (linear mixed-effects model with mouse as a random effect, ∗∗∗*p* = 0.001). (C) mEPSC amplitude in WT and eIF4E-TG mice (linear mixed-effects model with mouse as a random effect, ∗*p* = 0.0439). (D–F) Recordings were collected from 8 WT and 5 TG mice (neurons *n* = 17 WT and *n* = 16 TG). (D) Representative mIPSC traces recorded in presence of 1 μM TTX, 40 μM DL-AP5, and 10 μM DNQX (scale bars: 20 pA, 200 ms). (E) mIPSC frequency in WT and eIF4E-TG mice. (F) mIPSC amplitude in WT and eIF4E-TG mice (linear mixed-effects model with mouse as a random effect, ∗∗*p* = 0.0025). (G–I) Recordings were collected from 5 WT and 6 TG mice (neurons *n* = 17 WT and *n* = 15 TG). (G) Representative NMDA-mEPSC traces recorded in presence of 300 nM TTX, 50 μM picrotoxin, and 10 μM DNQX. Bottom trace shows disappearance of events following bath application of 10 μM AP5 confirming NMDA mediation (scale bars: 10 pA, 200 ms). (H) NMDA-mEPSC frequency in WT and eIF4E-TG mice (linear mixed-effects model with mouse as a random effect, ∗*p* = 0.0255). (I) NMDA-mEPSC amplitude in WT and eIF4E-TG mice. Data are presented as median ± quartiles within the boxplot, with whiskers indicating minimum and maximum values. Dots represent individual neurons. Statistical comparisons were performed using linear mixed-effects models with mouse as a random effect and degrees of freedom estimated using the Kenward-Roger method. Significance is denoted as ∗*p* < 0.05, ∗∗*p* < 0.01, and ∗∗∗*p* < 0.001. For full details of statistical analysis, including negative results, refer to Table S2.

To isolate AMPA receptor-mediated mEPSCs, recordings were performed at −70 mV in the presence of tetrodotoxin (TTX) to block sodium channels and prevent action potentials, and the GABAA-receptor antagonist picrotoxin to block inhibitory transmission. We found an increase in AMPA-mEPSC frequency and reduction in amplitude in eIF4E-TG mice (Figures 2A–2C and Table S2), suggesting dysregulated spontaneous excitatory transmission.We next recorded mIPSCs in the presence of TTX, as well as AMPA-receptor and NMDAR antagonists DNQX and AP5, using high-chloride internal solution to observe inward GABAergic currents at −70 mV. We found that while the mIPSC amplitude was reduced in eIF4E-TG SPNs, mIPSC frequency was unchanged (Figures 2D–2F and Table S2), suggesting an overall reduction in postsynaptic GABAergic signaling without changes in presynaptic inhibitory input. Taken together, these results indicate the presence of an excitatory-inhibitory imbalance in the inputs onto SPNs.

The increase in mEPSC frequency may reflect either an increase in presynaptic glutamate release or an increased number of excitatory synapses, whereas reduction in mEPSC amplitude is consistent with altered postsynaptic AMPA receptor-mediated currents. As glutamatergic signaling is mediated by both AMPARs and NMDARs, and it is possible to measure also spontaneous NMDAR-mediated transmission in SPNs,^75^ we then performed recordings in the presence of TTX, picrotoxin, and AMPAR-antagonist DNQX and in Mg^2+^-free artificial cerebrospinal fluid (ACSF) to unblock NMDARs at −70 mV. We found that the NMDA-mEPSC frequency was also enhanced in eIF4E-TG SPNs, while NMDA-mEPSC amplitude was unchanged (Figures 2G–2I and Table S2). Together, these data demonstrate increased frequency of AMPAR- and NMDAR-mediated mEPSCs, as well as reduced amplitude of AMPAR-mediated mEPSCs and GABAR-mediated mIPSCs in SPNs of eIF4E-TG mice, indicating altered spontaneous excitatory and inhibitory synaptic transmission.

### Unaltered corticostriatal connectivity

Our findings of altered spontaneous excitatory transmission in SPNs raised the possibility that excitatory input to the striatum may be enhanced, either globally or in an input-specific manner. The striatum primarily receives excitatory synaptic input from the cortex.^76^^,^^77^^,^^78^ Therefore, we sought to determine whether synaptic strength at defined corticostriatal inputs is altered in eIF4E-TG mice.

We targeted major sensorimotor cortical inputs to the dorsal striatum, including motor and somatosensory cortical regions.^79^ To selectively stimulate defined corticostriatal inputs, AAV-hSyn-hChR2(H134R)-mCherry was bilaterally injected into the target cortical regions. Mice were allowed to recover for at least 3 weeks before preparation of acute striatal slices for whole-cell recordings. Photostimulation was then used to activate channelrhodopsin-expressing sensorimotor corticostriatal axon terminals within the striatum.^80^

**Figure 3:**
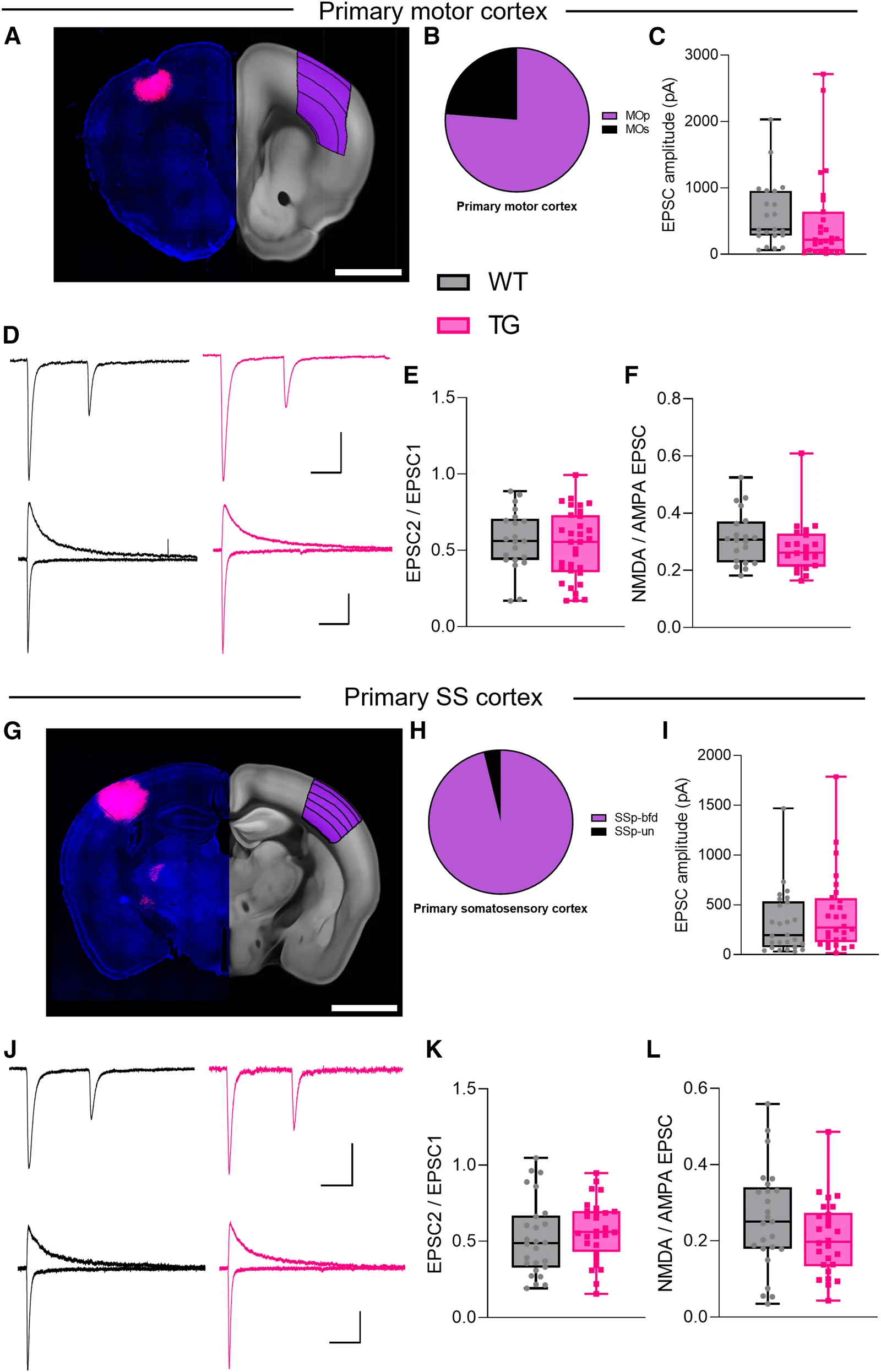
Optogenetic stimulation of dorsal striatal inputs in WT and eIF4E-TG mice (A) Left: example image of injection sites with mCherry expression (red) in a coronal brain slice at the level of the primary motor cortex, shown with DAPI neuronal staining (blue) (scale bars, 2 mm). Right: equivalent coronal section from the Allen Brain Atlas, with the target region indicated in purple. (B) Pie chart depicting the distribution of viral expression across cortical regions. MOp = primary motor cortex and MOs = secondary motor cortex. (C–F) Recordings were collected from 4 WT and 7 TG mice (PPR: neurons *n* = 21 WT and *n* = 32 TG, NMDA:AMPA neurons *n* = 20 WT and *n* = 21 TG). (C) Single pulse EPSC amplitude in WT and eIF4E-TG mice with optogenetic stimulation of motor corticostriatal axon terminals. (D) Example traces showing paired-pulse EPSCs and NMDA:AMPA EPSCs from WT and TG mice (top: paired-pulse scale bars: 100 pA, 50 ms; bottom: NMDA:AMPA scale bars: 100 pA, 100 ms). (E) Paired-pulse ratio in WT and eIF4E-TG mice. (F) NMDA:AMPA ratio in WT and eIF4E-TG mice. (G) Left: example image of injection sites with mCherry expression (red) in a coronal brain slice at the level of the primary somatosensory cortex, shown with DAPI neuronal staining (blue) (scale bars, 2 mm). Right: equivalent coronal section from the Allen Brain Atlas, with the target region indicated in purple. (H) Pie chart depicting the distribution of viral expression across cortical regions. SSp-bfd = primary somatosensory area, barrel field; SSp-un = primary somatosensory area, unassigned. (I–L) Recordings were collected from 9 WT and 7 TG mice (PPR: neurons *n* = 26 WT and *n* = 28 TG, NMDA:AMPA neurons *n* = 25 WT and *n* = 26 TG). (I) Single-pulse EPSC amplitude in WT and eIF4E-TG mice with optogenetic stimulation of somatosensory cortical axon terminals. (J) Example traces showing paired-pulse EPSCs and NMDA:AMPA EPSCs from WT and TG mice (top: paired-pulse scale bars: 100 pA, 50 ms; bottom: NMDA:AMPA scale bars: 100 pA, 100 ms). (K) Paired-pulse ratio in WT and eIF4E-TG mice. (L) NMDA:AMPA ratio in WT and eIF4E-TG mice. All recordings were performed in the presence of 50 μM picrotoxin to block GABAergic signaling. To account for the nested structure of the data, all statistical comparisons were performed using linear mixed-model (LMM) analysis, with mouse as a random effect, and degrees of freedom estimated using the Kenward-Roger method. For full details of statistical analysis, including negative results, refer to Table S3.

Targeting specificity was assessed by examining mCherry expression in cortical regions using the Napari plug-in DMC-BrainMap.^81^ In the motor cortex-injected mice, most viral expression was primarily localized to the targeted motor cortical regions, with limited spread to adjacent cortical areas (Figures 3A and 3B). Because these regions provide major excitatory input to the dorsal striatum, recordings were analyzed together as motor corticostriatal inputs. Optogenetic stimulation of corticostriatal axon terminals reliably elicited optogenetic EPSCs (oEPSCs) in SPNs (Figure 3C). When applying a set-intensity light stimulus, we observed no change in the oEPSC amplitude for motor-corticostriatal synapses (Figure 3C). We also found no change in paired pulse ratio or NMDA:AMPA ratio of oEPSCs induced at motor corticostriatal synapses (Figures 3D–3F). Statistical analyses accounted for clustering of recordings within mice using linear mixed-effects models with mouse identity as a random effect (Table S3).

Similarly, injections targeting somatosensory cortex resulted in expression predominantly restricted to somatosensory cortical regions (Figures 3G and 3H). We reliably elicited oEPSCs in SPNs with light stimulation of S1 terminals (Figure 3I) but observed no change in the oEPSC amplitude in eIF4E-TG mice (Figure 3I). We also found no change in paired pulse ratio or NMDA:AMPA ratio of oEPSCs at S1-SPN synapses (Figures 3J–3L). These findings indicate that glutamatergic synaptic strength and short-term presynaptic release properties sampled from motor and somatosensory corticostriatal inputs are largely preserved in eIF4E-TG mice under the conditions tested. Together, these findings suggest that increased spontaneous excitatory activity in eIF4E-TG SPNs is not accompanied by detectable alterations in baseline evoked transmission at major sensorimotor corticostriatal inputs.

### Enhanced LTP

**Figure 4:**
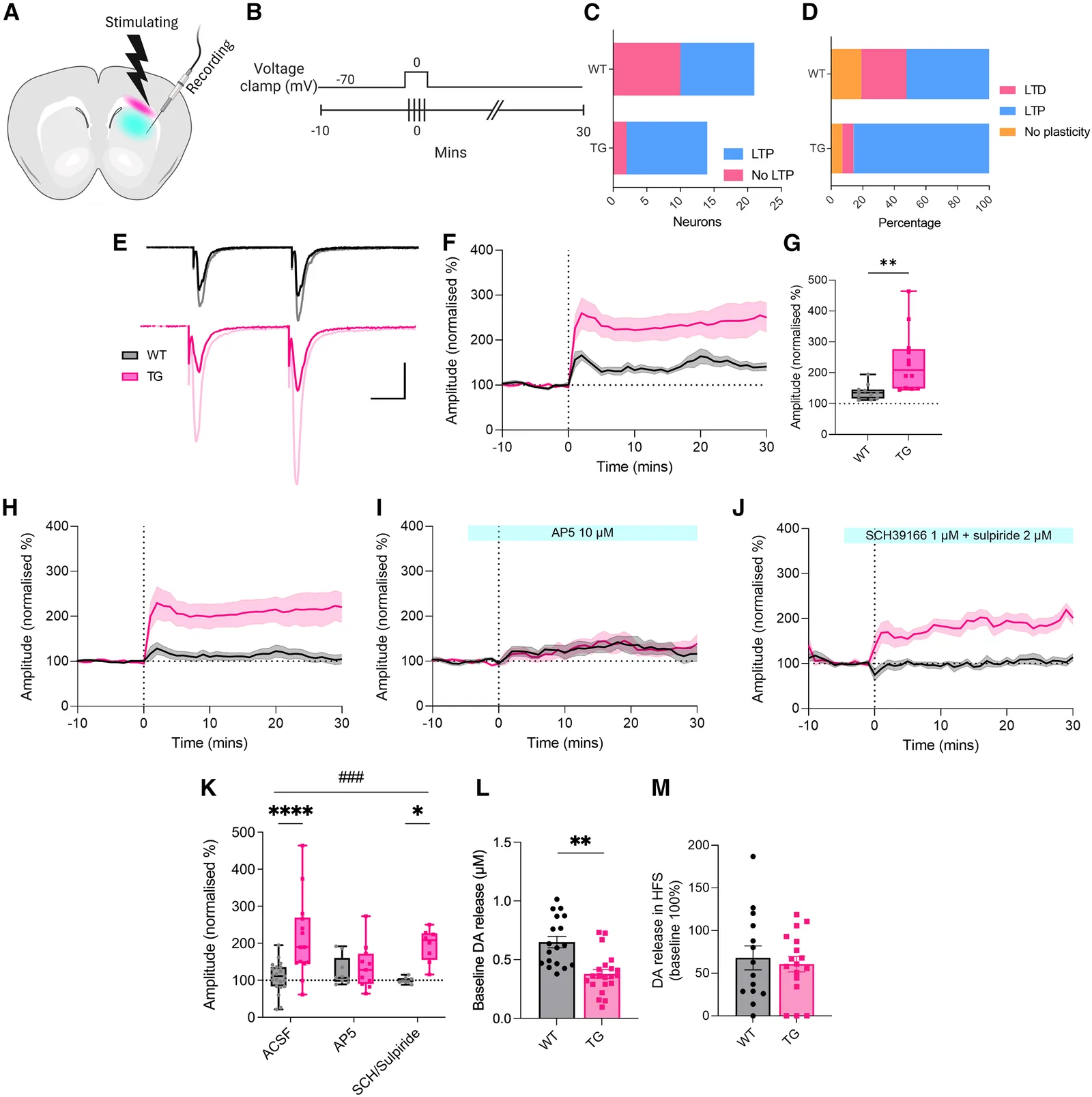
Plasticity induction with a high-frequency stimulation protocol in dorsal striatum of WT and eIF4E-TG mice (A) Schematic showing the stimulating and recording positions in the dorsal striatum. Region used for stimulation (within corpus callosum) is depicted in magenta, and region for recording (within the dorsal striatum) is depicted in cyan. (B) Schematic showing the stimulation protocol, with postsynaptic depolarization paired with electrical high-frequency stimulation (HFS). Baseline was established over a 10-min period prior to HFS, with paired EPSC (50 ms inter-spike interval) delivered every 20 s, and EPSCs were recorded up to 30 min post-HFS. HFS was given at 100 Hz for 1 s, repeated four times with 10-s intervals. Voltage was clamped at −70 mV throughout recording but increased to 0 mV during each bout of HFS. (C–K) In all recordings, baseline is normalized to 100%, and plasticity was quantified from the final 5 min of recording (25–30 mins), compared to the final 5 min of baseline (5–0 mins). Plasticity was defined as LTD (<90%), LTP (>110%), or showing no change in EPSC amplitude from baseline (>90% and <110%). (C) Proportion of cells exhibiting LTP (chi-squared test: X^2^ = 4.413, *p* = 0.0418). (D) Plasticity distribution: percentage of neurons expressing LTD, LTP, or no change from baseline. (E) Example traces showing paired-pulse EPSCs from LTP-expressing neurons from WT mice before (black) and after (gray) HFS, and from TG mice before (dark pink) and after (pale pink) HFS (scale bars: 200 pA, 20 ms). (F) EPSC amplitude (normalized to baseline 100%) for the duration of the recording period in cells expressing LTP (>110% post-stimulation) from WT and TG mice. Recordings were collected from 9 WT and 9 TG mice (neurons *n* = 11 WT, *n* = 12 TG). (G) EPSC amplitude 25–30 min after HFS (normalized to baseline 100%) in cells expressing LTP (linear mixed-model [LMM] analysis with mouse as a random effect, Kenward-Roger method, *t* [15.9] = −2.951; ∗∗*p* = 0.0094). (H) EPSC amplitude for the duration of the recording period in all cells from WT and TG mice in standard conditions (ACSF). ACSF recordings were collected from 17 WT and 10 TG mice (neurons *n* = 21 WT and *n* = 14 TG). (I) EPSC amplitude for the duration of the recording period in all cells from WT and TG mice following bath application of 10 μM D-AP5. D-AP5 recordings were collected from 7 WT and 8 TG mice (neurons *n* = 9 WT and *n* = 11 TG). (J) EPSC amplitude for the duration of the recording period in all cells from WT and TG mice following bath application of 1 μM SCH39166 and 2 μM sulpiride. SCH/sulpiride recordings were collected from 4 WT and 4 TG mice (neurons *n* = 8 WT and *n* = 8 TG). (K) EPSC amplitude in all neurons from WT and TG mice, recorded in ACSF, D-AP5, or SCH39166/sulpiride. There was a nonsignificant trend observed in the group × condition interaction (F [2, 41.1] = 2.84, *p* = 0.070) with a significant effect of genotype (F [1, 36.5] = 15.19, ###*p* = 0.0004, LMM with mouse as random effect, Kenward-Roger method). Pairwise comparisons revealed significant TG vs. WT differences under ACSF (*t* [47.4] = 4.34, ∗∗∗∗*p* < 0.0001) and sulpiride conditions (*t* [28.4] = 2.54, ∗*p* = 0.017, Tukey-corrected), but not under AP5 (*t* [52.4] = 0.46, *p* = 0.64). (L and M) Fast-scan cyclic voltammetry recordings were obtained from 6 WT and 6 TG mice (slices *n* = 18 WT and *n* = 21 TG). A detailed breakdown of statistics is presented in Table S4. (L) Baseline dopamine (DA) release evoked with electrical stimulation (LMM analysis with mouse as a random effect, Kenward-Roger method, *t* [9.81] = −3.321, ∗∗*p* = 0.0079). (M) DA release during HFS, expressed as a percentage of baseline release (100%). All recordings depicted in (A)–(K) were performed in the presence of 50 μM picrotoxin to block GABAergic signaling. Data are presented as median ± quartiles within the boxplot, with whiskers indicating minimum and maximum values (G and K), as mean ± SEM, with the mean represented by a connecting line and SEM by error lines with shading (F and H–J), or as mean ± SEM, with bars indicating the mean and error bars depicting SEM (L and M). Dots represent individual values for each neuron/slice. (D) and (F–J) illustrate the time course of recordings; statistical analyses were performed on datasets quantified in (C), (G), (K), (L), and (M). Statistical comparisons were performed using chi-squared tests, LMMs with mouse as a random effect, or two-way ANOVA, where appropriate. Significance is denoted as ∗*p* < 0.05, ∗∗*p* < 0.01, and ∗∗∗∗*p* < 0.0001. For full details of statistical analysis, including negative results, refer to Table S4.

Previous studies in eIF4E-TG- and 4E-BP2-knockout mice have reported dysregulated forms of synaptic plasticity, including enhanced hippocampal LTP^82^ and greater striatal LTD.^70^ In addition, we recently identified reduced striatal dopamine release in eIF4E-TG mice.^68^ Based on our observations of increased dendritic spine density, enhanced frequency of AMPAR- and NMDAR-mediated mEPSCs, and reduced AMPAR-mEPSC amplitude, we hypothesized that striatal SPNs in eIF4E-TG mice might display an altered plasticity profile. To test this, we performed whole-cell recordings with electrical stimulation of the white matter directly above the dorsal striatum (Figure 4A). We used a high-frequency stimulation (HFS) protocol adapted from previous studies^83^^,^^84^^,^^85^ consisting of four trains of 100 Hz stimulation (1-s duration and 10-s interval), each paired with postsynaptic depolarization to 0 mV (Figure 4B).

In all experiments, LTP was defined as >10% increase from baseline EPSC amplitude (>110%) measured 30 min after stimulation, and LTD as >10% reduction (<90%). Our stimulation protocol induced LTP in both WT and TG mice, but we found an increased proportion of LTP-expressing neurons in the eIF4E-TG mice compared to WT (Figure 4C). In WT mice, half of the recorded neurons exhibited LTP, with roughly a quarter showing LTD and a quarter showing no change from baseline, whereas over 80% of neurons in TG mice expressed LTP (Figure 4D). When analyzing only LTP-expressing neurons, eIF4E-TG SPNs exhibited greater post-stimulation EPSC amplitude compared to WT neurons (Figures 4E–4G), suggesting an increase in both the likelihood and in the magnitude of LTP. When all recorded neurons were included, WT neurons displayed bidirectional plasticity with no significant net change, whereas TG neurons showed an overall shift toward potentiation (Figures 4H and 4K), resulting in a significant genotype difference in post-stimulation EPSC amplitude (Figure 4K). Together, these findings indicate that eIF4E-TG SPNs exhibit a bias toward potentiation under these recording conditions. As NMDARs are vital for the induction of many forms of LTP, including in the striatum,^8^^,^^48^^,^^49^ we sought to determine whether this form of LTP also exhibits NMDAR dependency. The NMDAR antagonist AP5 (10 μM) was bath applied during baseline acquisition and HFS induction. Under these conditions, no difference in net plasticity was observed between WT or TG neurons (Figures 4I and 4K), suggesting that LTP induction in this paradigm requires NMDAR activation.

Because dopamine signaling is critical to many forms of striatal plasticity,^12^^,^^48^^,^^52^^,^^53^^,^^54^ we next tested the effect of dopamine receptor antagonism by bath applying the D1R antagonist SCH39166 (1 μM) and the D2R antagonist sulpiride (2 μM). Under these conditions, TG neurons continued to show net potentiation (Figures 4J and 4K), whereas WT neurons did not exhibit a consistent shift toward either potentiation or depression (Figure 4K). Overall, these findings indicate that LTP in eIF4E-TG SPNs was sensitive to NMDAR blockade but was not prevented by D1R/D2R antagonism under the conditions tested.

As we previously observed reduced evoked dopamine release in the dorsal striatum of the TG mice,^68^ we asked whether altered dopamine dynamics during HFS could account for the persistence of LTP under dopamine receptor antagonism. Therefore, we used fast-scan cyclic voltammetry to record electrically evoked dopamine release in acute striatal slices during the HFS protocol described above. In line with our previous findings, baseline dopamine release was significantly reduced in eIF4E-TG mice (Figure 4L and Table S4). Dopamine release during HFS was detectable and did not differ from WT when expressed relative to baseline (Figure 4M). This finding suggests that although baseline dopamine release is reduced in the eIF4E-TG mice, dopamine release during HFS is preserved and does not readily explain the continued expression of LTP in the presence of dopamine receptor antagonists.

### Altered somatodendritic Ca^2+^ dynamics in eIF4E-TG SPNs

Synaptic plasticity in SPNs is strongly influenced by coordinated dopamine-dependent cAMP signaling and NMDAR-mediated Ca^2+^ influx.^86^^,^^87^^,^^88^ As LTP in eIF4E-TG SPNs persisted under dopamine receptor antagonism, we asked whether depolarization-evoked Ca^2+^ dynamics are altered in these neurons. We therefore used the genetically encoded Ca^2+^ indicator GCaMP7s and 2-photon (2P) microscopy to measure somatic and dendritic Ca^2+^ signals in SPNs from acute striatal slices following brief somatic depolarization.

AAV-*syn*-FLEX-jGCaMP7s-WPRE was bilaterally injected into the striatum of Adora2a-Cre/WT and -/eIF4E-TG or D1-Cre/WT and -/eIF4E-TG mice. After 3 weeks of recovery, acute striatal slices were prepared for imaging. SPNs were identified by baseline GCaMP7s fluorescence and voltage clamped at −70 mV in whole-cell configuration. Cells were briefly depolarized to 0 mV in the absence of HFS to examine depolarization-evoked postsynaptic Ca^2+^ responses.

**Figure 5:**
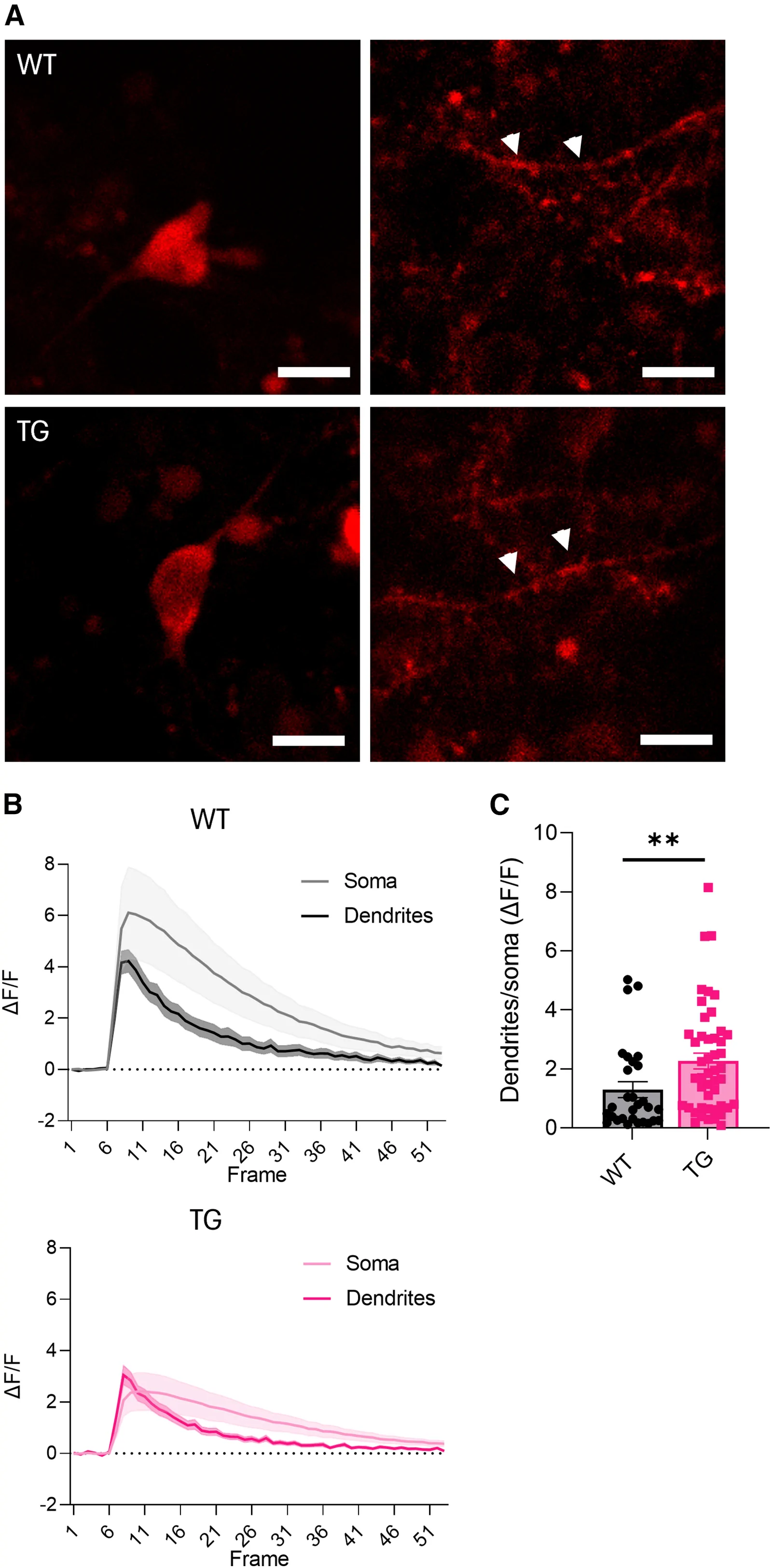
Calcium dynamics in SPNs of WT and eIF4E-TG mice (A) Representative images of GCaMP7s activity in soma and in dendrites (60 μm away from soma) during depolarization of SPNs (scale bars, 10 μm). White arrows indicate dendritic signal observed during depolarization. (B) Time course of GCaMP7s activity in soma and dendrites of WT (left) and TG (right). Recordings were collected from 4 WT and 5 TG mice (neurons *n* = 11 WT and *n* = 16 TG; dendrites *n* = 30 WT, *n* = 47 TG). (C) Dendrite-to-soma ratio of depolarization-evoked GCaMP7s peak amplitude (Mann-Whitney test: U = 422, ∗∗*p* = 0.0051). Data are presented as mean ± SEM, with the mean represented by a connecting line and SEM by error lines with shading (B), or as mean ± SEM, with bars indicating the mean and error bars depicting SEM (C). Significance is denoted as ∗*p* < 0.05, ∗∗*p* < 0.01, and ∗∗∗∗*p* < 0.0001, calculated using the Mann-Whitney test. (B) illustrates the time course of depolarization-evoked Ca^2+^ responses; statistical analyses were performed on datasets quantified in (C). For full details of statistical analysis, including negative results, refer to Table S5.

Somatic and dendritic Ca^2+^ signals were imaged, with dendritic regions analyzed 60 μm from the soma. Depolarization-evoked GCaMP7s signals were reduced in both soma and dendrites of eIF4E-TG SPNs compared to WT (Figures 5A and 5B). This effect was observed in both D1-and Adora2a-expressing cells (data not shown). As absolute GCaMP fluorescence can vary across preparations, we additionally analyzed dendritic responses relative to somatic responses within the same neuron. When expressed as a dendrite-to-soma ratio, Ca^2+^ signals were significantly increased in TG neurons (Figure 5C), indicating an altered spatial distribution of depolarization-evoked Ca^2+^ dynamics. Because both somatic and dendritic signals were reduced in TG neurons, the altered dendrite-to-soma ratio cannot distinguish between proportionally greater somatic reduction, relatively preserved or greater dendritic signaling, or a combination of both. Together, these findings demonstrate altered compartmental Ca^2+^ signaling in eIF4E-TG SPNs during depolarization, which may influence synaptic integration during plasticity-inducing conditions.

## Discussion

In this study, we identify an altered striatal synaptic phenotype in eIF4E-TG mice characterized by increased dendritic spine density, changes in spontaneous excitatory and inhibitory transmission, and enhanced NMDAR-dependent potentiation induced by HFS. Although baseline synaptic strength at major sensorimotor corticostriatal inputs was preserved, SPNs from TG mice displayed an increased likelihood and magnitude of LTP under disinhibited recording conditions. Notably, this potentiation was sensitive to NMDAR blockade but was not prevented by D1/D2-receptor antagonism at the concentrations tested. In parallel, we observed altered compartmental Ca^2+^ dynamics during postsynaptic depolarization, suggesting that eIF4E overexpression modifies both structural and activity-dependent properties of striatal SPNs.

### Altered excitatory and inhibitory synaptic transmission

We observed an increase in dendritic spine density accompanied by an increase in mEPSC frequency, consistent with an increased number of excitatory synaptic contacts onto SPNs. The elevation in mEPSC frequency was detected for both AMPAR- and NMDAR-mediated events, indicating enhanced baseline glutamate signaling. Increased mEPSC frequency can reflect either higher presynaptic release probability or an increased number of functional synapses; however, the parallel increase in dendritic spine density supports the possibility of increased synapse number in eIF4E-TG SPNs.

Similar increases in spine density and mEPSC frequency have been reported in other syndromic models of ASD characterized by elevated mTORC1-signaling and neuronal protein synthesis, including FXS mice, although in that case the effect was restricted to D1-SPNs.^26^ Increased mEPSC frequency has also been observed across multiple ASD models,^89^^,^^90^ suggesting that elevated spontaneous excitatory transmission may represent a convergent phenotype.

In contrast, we observed a reduction in AMPA-mEPSC amplitude, which may indicate altered postsynaptic AMPAR function or composition. Together with the altered distribution of spine subtypes, these findings suggest changes in excitatory synaptic organization rather than a simple increase in synapse number. Consistent with this interpretation, FXS mice show dysregulated AMPAR GluR1/2 subunit expression^91^ and immature striatal dendritic spine morphology,^60^ suggesting that increased synapse number may coexist with altered synaptic maturation in mTORC1-related ASD models.

We also identified a reduction in mIPSC amplitude without a change in mIPSC frequency in the eIF4E-TG mice. While this pattern is consistent with altered postsynaptic GABAergic signaling, our data do not provide direct evidence regarding changes in inhibitory synapse number or presynaptic release probability. Together, the combined alterations in spontaneous excitatory and inhibitory transmission are compatible with a shift in excitatory-inhibitory balance in the striatum of the eIF4E-TG mice. Similar alterations in striatal excitatory-inhibitory balance have been reported in various ASD models including FXS mice^40^^,^^92^^,^^93^ as well as clinical studies.^93^ However, because corticostriatal and plasticity recordings were performed in the presence of picrotoxin, the functional contribution of altered inhibitory signaling to synaptic plasticity in the striatum of the eIF4E-TG mice remains to be determined. Future work should examine potential changes in striatonigral, striatopallidal, and intrastriatal inhibitory transmission.

### Preserved corticostriatal connectivity

Altered corticostriatal connectivity has been widely associated with various forms of ASD, both in clinical^55^^,^^58^^,^^94^^,^^95^^,^^96^^,^^97^^,^^98^^,^^99^ and preclinical studies.^26^^,^^55^^,^^56^^,^^57^^,^^58^^,^^100^ Besides the increase in spontaneous excitatory transmission, we did not detect changes in paired-pulse ratio or NMDA:AMPA ratio at motor or somatosensory corticostriatal inputs. These findings indicate that the major sensorimotor corticostriatal synapses exhibit preserved baseline transmission in eIF4E-TG mice. However, our experiments were restricted to defined sensorimotor inputs, and other cortical or subcortical afferents to the striatum were not examined. In addition, an increase in the number of synaptic contacts would not necessarily be reflected in measures of synaptic strength during evoked stimuli, which produces synchronized glutamate release from multiple synapses. Our results suggest that enhanced spine density and mEPSC frequency may coexist with preserved evoked synaptic strength at specific input pathways.

### Dysregulated plasticity in the striatum

SPNs express bidirectional plasticity in both D1-and D2-SPNs.^101^^,^^102^^,^^103^ Although LTD is often considered the preponderant form of plasticity in the striatum,^4^^,^^50^^,^^104^ the direction of plasticity depends strongly on induction protocol and cellular context. In extracellular recordings without paired depolarization, HFS principally triggers striatal LTD,^4^ whereas coincident postsynaptic depolarization that relieves NMDAR Mg^2+^ block favors LTP.^105^ White matter stimulation has been shown to preferentially drive striatal LTP expression under such paired condition.^48^ In addition, LTP is more readily triggered in *in vivo* conditions.^106^ Thus, the induction protocol and local microenvironment critically determine the direction of plasticity expressed by SPNs.

Under our whole-cell paired HFS protocol, both LTP and LTD were observed in WT neurons, reflecting bidirectional plasticity. In contrast, eIF4E-TG SPNs exhibited a marked bias toward LTP, with an increased proportion of neurons expressing potentiation and an enhanced magnitude of LTP. These findings indicate that eIF4E overexpression shifts plasticity rules toward potentiation under these recording conditions.

The enhanced potentiation observed here extends previous findings of exaggerated striatal LTD in eIF4E-TG mice obtained using extracellular field recordings.^70^ However, the two studies used substantially different induction paradigms and recording configurations. Whereas the previous study employed extracellular field recordings without paired postsynaptic depolarization, the current experiments used whole-cell recordings with postsynaptic depolarization during HFS, a protocol capable of producing either potentiation or depression depending on the cellular state and pattern of activity, and expected to strongly engage postsynaptic NMDAR-dependent Ca^2+^ signaling. Together, these findings suggest that eIF4E overexpression alters the rules governing striatal synaptic plasticity in a state- and protocol-dependent manner rather than producing a uniform shift toward either potentiation or depression.

Several mechanisms may contribute to this shift. The increased number of dendritic spines and elevated mEPSC frequency suggest a higher density of excitatory synapses, which may lower the threshold for potentiation. In addition, the reduction in AMPAR-mediated mEPSC amplitude raises the possibility that synapses may have reduced baseline AMPAR content, potentially allowing greater dynamic range for AMPAR insertion during LTP. The unchanged NMDA-mEPSC amplitude suggests that NMDAR-mediated currents are preserved, potentially altering the relative contribution of NMDAR versus AMPAR signaling during plasticity induction.

Importantly, the observed phenotype is not fully explained by a simple increase in NMDAR-mediated transmission, as NMDA-mEPSC amplitude was unchanged. Instead, the combination of altered synaptic structure, AMPAR function, and dendritic Ca^2+^ dynamics suggests a modification of synaptic integration and plasticity thresholds.

Importantly, blockade of NMDARs abolished LTP in both genotypes, indicating that this form of potentiation remains NMDAR-dependent. Thus, the enhanced LTP observed in eIF4E-TG SPNs does not represent a novel induction mechanism, but rather a shift in the probability and magnitude of NMDAR-dependent potentiation, biasing the outcome of synaptic plasticity toward potentiation under defined activity conditions.

### Dopamine dependence and NMDAR gating of plasticity

Although dopamine release is reduced in the eIF4E-TG mice,^68^ it is still observed during HFS stimulation. The persistence of LTP in the presence of D1R and D2R antagonists indicates that, under our experimental conditions, potentiation is not prevented by canonical dopaminergic receptor signaling. This suggests that eIF4E overexpression may shift plasticity toward a postsynaptically driven, NMDAR-dependent form of potentiation with reduced dependence on dopamine receptor activation.

Interestingly, it was found that knockout of D2 receptors can reveal an HFS-induced, NMDAR-dependent LTP in striatal slices that is insensitive to D1R/D2R blockade.^107^ In WT mice, the same HFS protocol induces LTD and only induces LTP in Mg^2+^-free ACSF, and both directions are modifiable by D1/D2R antagonism.^107^ The LTP phenotype in D2R-knockout mice may be driven by homeostatic alterations in NMDAR and glutamatergic signaling and potentially reflects the role of D2R activation in inhibition of Ca^2+^ currents,^107^^,^^108^ which are important for LTP expression in the striatum.^109^ It is therefore possible that altered dopamine receptor signaling may shift the balance of Ca^2+^-dependent plasticity mechanisms. However, because enhanced LTP was observed in both D1-and D2-SPNs, a mechanism affecting NMDAR-mediated signaling more broadly may be more consistent with our data.

The role of dopamine in striatal LTP has also been studied in Parkinsonian mouse models. Although Parkinsonian models such as Parkin- and PINK1-knockout mice exhibit impaired HFS-induced LTP or LTD in the context of reduced striatal dopamine release,^63^^,^^110^ eIF4E-TG mice display enhanced striatal plasticity^17^ despite reduced dopamine signaling.^68^ These opposing phenotypes may reflect differences in the underlying molecular perturbation. Parkin and PINK1 regulate protein turnover pathways, including components of the ubiquitin-proteasome system, autophagy, and mitophagy,^111^^,^^112^ whereas eIF4E overexpression selectively enhances cap-dependent translation initiation.^113^ Because eIF4E preferentially enhances translation of specific mRNA subsets, including transcripts involved in synaptic structure and function, altered translational selectivity, rather than generalized protein accumulation, may reshape the synaptic proteome of SPNs. Thus, differential regulation of protein synthesis and degradation may distinctly influence striatal plasticity outcomes. The balance between translational control and protein turnover may therefore be critical for maintaining normal plasticity in the striatum.

Although protein synthesis has been extensively linked to synaptic plasticity in hippocampal circuits, its contribution to striatal plasticity remains comparatively less characterized.^18^^,^^66^^,^^82^ We previously demonstrated increased rates of protein synthesis in the striatum of eIF4E-TG mice using the SUnSET assay.^69^ While the present study does not directly test translation dependence during LTP induction or maintenance, the enhanced probability and magnitude of LTP observed here are consistent with a shift in plasticity thresholds that may arise from altered translational control. Such changes may facilitate stabilization of potentiated synapses once induced, thereby biasing SPNs toward persistent potentiation. These findings extend the framework of translational regulation into striatal plasticity phenotypes relevant to neurodevelopmental disorders.

Striatal plasticity requires coordinated NMDAR-mediated Ca^2+^ influx^86^^,^^88^^,^^109^ together with dopamine-dependent signaling.^12^^,^^52^^,^^53^^,^^54^^,^^102^^,^^114^^,^^115^ In our recordings, postsynaptic depolarization during HFS would relieve the voltage-dependent Mg^2+^ block of NMDARs, promoting Ca^2+^ entry and favoring LTP induction.^105^^,^^116^ Although WT SPNs expressed bidirectional plasticity under this protocol, eIF4E-TG SPNs showed a bias toward potentiation that remained strictly NMDAR-dependent.

Given the established role of NMDAR-mediated dendritic Ca^2+^ microdomains in regulating synaptic plasticity,^87^ we examined whether Ca^2+^ signaling was altered in TG neurons. While absolute depolarization-evoked Ca^2+^ signals were reduced in TG SPNs, the relative dendrite-to-soma Ca^2+^ ratio was increased indicating altered compartmental distribution of Ca^2+^ signals.

While the altered dendrite-to-soma ratio does not by itself resolve whether the effect reflects relatively greater dendritic responses, relatively reduced somatic responses, or a combination of both (see results), the findings nevertheless support altered distribution of depolarization-evoked Ca^2+^ signaling between dendrites and soma in TG SPNs. Previous control experiments performed using the same GCaMP7s viral strategy in eIF4E-TG mice did not reveal genotype-dependent differences in GCaMP7s expression levels assessed by western blot analysis,^69^ suggesting that differences in reporter expression are unlikely to account for the altered Ca^2+^ dynamics observed here. Because dendrites represent the principal site of synaptic integration and LTP expression, such redistribution may lower the threshold for potentiation without requiring an overall increase in global Ca^2+^ levels. Notably, NMDAR activation can under certain conditions drive sustained Ca^2+^ elevations that support dopamine-independent LTP,^117^ suggesting that sufficient postsynaptic Ca^2+^ signaling may overcome canonical dopamine dependence. Importantly, we did not observe genotype-dependent differences in passive membrane properties (Table S9) in either the LTP or NMDA-mEPSC datasets, suggesting that the altered plasticity and Ca^2+^ signaling phenotypes are unlikely to reflect gross differences in baseline neuronal properties or recording stability under the conditions tested.

Translational dysregulation has been shown to impact NMDAR function in other ASD-related models, such as FXS mice, where altered NMDAR currents are associated with disrupted striatal LTP.^118^ Together, these findings raise the possibility that altered dendritic Ca^2+^ integration in eIF4E-TG SPNs contributes to the observed bias toward NMDAR-dependent, dopamine-insensitive potentiation. Future studies will be required to determine whether the exaggerated LTD previously observed in this model^70^ similarly exhibits altered dopamine dependence and how compartmental Ca^2+^ dynamics interact with translational control to shape plasticity direction.

### Possible behavioral consequences of altered plasticity

As the induction protocol in our study generates LTP and LTD in WT neurons in roughly equal magnitudes and proportions, the average post-stimulation EPSC observed across neurons remained near baseline. This suggests that under our recording conditions, bidirectional plasticity in WT SPNs does not produce a net population-level shift in synaptic strength. In contrast, the bias toward potentiation observed in eIF4E-TG SPNs results in a net increase in excitatory drive at the population level. How such a shift translates to circuit and behavioral outcomes remains to be determined.

Corticostriatal plasticity is strongly linked to striatal-dependent learning processes.^119^ Striatal LTP is associated with both procedural learning and goal-directed learning,^85^^,^^120^^,^^121^^,^^122^^,^^123^ and synchronized SPN activity is particularly important for flexibility of goal-directed learning.^124^^,^^125^ A persistent bias toward potentiation, particularly under conditions of reduced dopamine sensitivity, may alter updating of learned associations when contingencies change,^126^^,^^127^ resulting in more rigid behavioral outcomes. Such a mechanism is consistent with previously reported impairments in reversal learning in eIF4E-TG mice^68^^,^^70^ and with broader evidence linking dopaminergic dysfunction to behavioral inflexibility in ASD models.^128^ However, direct causal links between the plasticity phenotype described here and behavioral outcomes remain to be established.

In conclusion, we identify an altered profile of striatal synaptic plasticity in eIF4E-TG mice characterized by increased spine density, enhanced spontaneous excitatory transmission, and a bias toward NMDAR-dependent potentiation. This potentiation was not prevented by D1/D2-receptor antagonism at the concentrations tested and was accompanied by altered compartmental Ca^2+^ dynamics. Together, these findings indicate that dysregulated translational control is associated with a shift in striatal plasticity rules toward NMDAR-dependent potentiation. Such changes may contribute to altered circuit function and behavioral inflexibility observed in this model, and highlight the importance of mRNA translational regulation in shaping striatal synaptic integration.

### Limitations of the study

The present study identifies an altered NMDAR-dependent plasticity profile in striatal projection neurons of eIF4E-TG mice; however, several limitations should be considered. First, although eIF4E overexpression increases translational capacity, protein synthesis was not directly manipulated during LTP induction or maintenance, and causality between altered translation and enhanced potentiation remains to be established. Second, plasticity experiments were conducted under disinhibited conditions with the addition of picrotoxin, and with single-concentration dopaminergic antagonism, limiting conclusions regarding dopamine dependence across physiological states. While the bath application of picrotoxin ensures minimal interference from inhibitory networks and removes the parameter of altered inhibitory connectivity in eIF4E-TG mice, this reduces the relevance to *in vivo* intact physiological conditions. Third, white-matter stimulation recruits heterogeneous glutamatergic afferents, precluding pathway-specific attribution of the observed LTP. Addressing these questions will require input-selective induction protocols, translation-targeted manipulations, and experiments under intact inhibitory control.

## Resource availability

### Lead contact

Requests for further information and resources should be directed to and will be fulfilled by the lead contact, Emanuela Santini (emanuela.santini@ki.se).

### Materials availability

This study did not generate new unique reagents.

### Data and code availability

• The data reported in this paper will be shared by the lead contact upon request.
• All original code used for linear mixed-effects model analyses is available in this paper’s supplemental information (Data S1). The corresponding data table arrangement used for these analyses is provided in Data S2.
• Any additional information required to reanalyse the data reported in this paper is available from the lead contact upon request.

## Acknowledgments

We thank the past and current members of Borgkvist and Santini labs for their methodological support and regular insightful discussions. We would like to thank Gilad Silberberg, Maya Ketzef, and other members of the neuroscience department for their considerate feedback and advice. We are grateful to Gilberto Fisone for providing the Tg(Drd2-EGFP)S118Gsat^+/−^ mice. We extend further thanks to the staff and veterinarians of the Comparative Medicine Biomedicum (KM-B) for their continuous assistance in maintaining the mouse colonies. This work was supported by the 10.13039/501100004063Knut and Alice Wallenberg Foundation (Wallenberg Academy Fellow Grant KAW 2017-0169 and project grant 2020-0054 to E.S.), the 10.13039/501100004359Swedish Research Council (2016-02758, 2023-02943 to E.S.; 2016-03129, 2024-03684 to A.B.), Olle Engkvist Stiftelse (E.S. and A.B.), Åhlén’s Foundation (A.B.), Magn. Bergvall’s Foundation (A.B.), The Strategic Research Program in Neuroscience (StratNeuro) starting (E.S. and A.B.) and bridging (E.S.) grants, 10.13039/501100004047Karolinska Institutet starting grant (E.S.).

## Author contributions

Conceptualization, A.A., A.B., and E.S.; methodology, A.A., A.B., and E.S.; validation, A.A., A.B., and E.S.; formal analysis, A.A., D.R.G., A.B., and E.S.; investigation, A.A., J.R, J.O., C.C., O.L., A.B., and E.S.; resources, A.B. and E.S.; writing – original draft, A.A., C.C., J.O., A.B., and E.S.; writing – review and editing, A.A., D.R.G., J.R., A.B., and E.S.; visualization, A.A, A.B., and E.S.; supervision, A.B. and E.S.; project administration, A.B. and E.S.; funding acquisition, E.S. and A.B.

## Declaration of interests

The authors report no biomedical financial interests or potential conflicts of interest.

## STAR★Methods

### Key resources table

**Table:** 

REAGENT or RESOURCE	SOURCE	IDENTIFIER
**Antibodies**		

Rabbit anti-Red Fluorescent Protein (RFP)	Rockland	Cat#600-401-379; RRID: AB_2209751
Goat anti-mouse Alexa Fluor 488	Thermo Fisher Scientific	Cat#A-11001; RRID: AB_2534069
Goat anti-rabbit Alexa Fluor 568	Thermo Fisher Scientific	Cat#A-11011; RRID: AB_143157
Streptavidin Alexa Fluor 488 Conjugate	Thermo Fisher Scientific	Cat# S11223; RRID: N/A

**Bacterialandvirusstrains**		

pAAV-hSyn-hChR2(H134R)-mCherry	Addgene; Karl Deisseroth	Addgene #26976; RRID:Addgene_26976
pGP-AAV-*syn*-FLEX-jGCaMP7s-WPRE	Addgene; Dana et al.^129^	Addgene #104491; RRID:Addgene_104491

**Chemicals,peptides, andrecombinantproteins**		

Tetrodotoxin (TTX)	HelloBio	Cat#HB1035
Picrotoxin	Tocris	Cat#1128
DNQX	HelloBio	Cat#HB0261
D-AP5	Tocris	Cat#0106
SCH39166	Tocris	Cat#2299
Sulpiride	Tocris	Cat#0895
Buprenorphine (Vetergesic Vet)	Apoteket	N/A
Carprofen (Rimadyl Vet)	Apoteket	N/A
Lucifer Yellow CH lithium salt	Invitrogen	Cat#L453
SeTau-647	Hölzel biotech	Cat#SETA-K9-4150

**Experimentalmodels:Organisms/strains**		

Mouse: eIF4E transgenic (eIF4Ewt/βtEif4e)	Ruggero et al.^130^	N/A
Mouse: Drd1a-TdTomato	Jackson Laboratory	Stock #016204; RRID: IMSR_JAX:016204
Mouse: Tg(Drd2-EGFP)S118Gsat/Mmnc	GENSAT/Rockefeller University	Stock#000230-UNC; RRID: MMRRC_000230-UNC

**Software andalgorithms**		

ZEN 3.4	Zeiss	https://www.zeiss.com/microscopy; RRID: N/A
pClamp 11	Molecular Devices	https://www.moleculardevices.com; RRID: SCR_011323
Clampfit	Molecular Devices	https://www.moleculardevices.com; RRID: N/A
Stimfit	Guzman et al.^131^	https://www.stimfit.org; RRID: SCR_016050
Demon Voltammetry Suite	Yorgason et al.^132^	https://github.com/DemonVoltammetry/Demon-Voltammetry-and-Analysis; RRID: N/A
ScanImage 2021.0.0.3baa4b74be	Vidrio Technologies	https://scanimage.org; RRID: N/A
WinWCP v5.8.3	University of Strathclyde	https://spider.science.strath.ac.uk/sipbs/software_ses.htm; RRID: N/A
ImageJ v1.54p	Schneider et al. XX	https://imagej.nih.gov/ij/; RRID: N/A
Imaris 9.5.1	Oxford Instruments	https://imaris.oxinst.com; RRID: N/A
DMC-BrainMAP Napari plugin	Jung et al.^81^	https://github.com/hejDMC/dmc-brainmap; RRID: SCR_027433
GraphPad Prism	GraphPad Software	https://www.graphpad.com; RRID: SCR_002798
R 4.6.0	R Foundation	https://www.r-project.org; RRID: N/A
lme4	CRAN	https://cran.r-project.org; RRID: SCR_015654
lmerTest	CRAN	https://cran.r-project.org; RRID: SCR_015656
Emmeans	CRAN	https://cran.r-project.org; RRID: SCR_018734

**Other**		

Multiclamp 700B amplifier	Molecular Devices	RRID: SCR_018455
Axon Digidata 1550B	Molecular Devices	N/A
Chem-Clamp 5MEG amplifier	Dagan Corporation	N/A
Scientifica Hyperscope multiphoton microscope	Scientifica	N/A

### Experimental model and study participant details

#### Animals

All experiments were compliant with the ethical permits issued by the Swedish Board of Agriculture (Ethical number: 18194-2018 and 19345-2023) and performed in accordance with the European Parliament and Council Directive 210&63/EU, 22nd September 2010 for protection of animals used for scientific purposes. Mice were sex-separated and group-housed (up to five mice per cage) in a temperature (23 °C) and humidity (55%) controlled environment, on a 12-h light/dark cycle with water and food available *ad libitum*. In all cases, eIF4E^wt/wt^ mice are referred to as wild type (WT), and eIF4E^wt/βtEif4e^ mice are referred to as eIF4E-transgenic (eIF4E-TG or TG).^70^^,^^130^ We used postnatal day 60–90 (2-3-month-old) male and female double-transgenic mice generated by crossing Drd1a-TdTomato^+/−^ (D1-TdTomato) mice^133^ (Jackson Laboratory, #016204) with eIF4E^wt/βtEif4e^ producing D1-TdTomato/WT and D1-TdTomato/TG, or by crossing Tg(Drd2-EGFP)S118Gsat/Mmnc^+/−^ (D2-eGFP) mice generated by the Gene Expression Nervous System Atlas (GENSAT) program at Rockefeller University^134^ with eIF4E^wt/βtEif4e^ producing D2-eGFP/WT and D2-eGFP/TG mice. Genotypes were confirmed by PCR as previously described.^68^^,^^69^ All lines were maintained in hemizygosity by crossing with C57BL/6J mice purchased from Janvier. Due to the absence of differences between the identified D1-and D2-SPNs in all parameters, results from both SPN types have been combined. In addition, no significant genotype X sex interactions were detected (Table S8), and data from males and females were therefore pooled for analysis.

### Method details

#### Stereotaxic surgery

Mice were injected with buprenorphine (i.p. 0.1 mg/kg) and anesthetized with 2% isoflurane. Adeno-associated virus (AAV) injections were performed via a Quintessential Stereotaxic Injector (Stoelting) at a rate of 30 nL/min. To assess the corticostriatal input strength, bilateral viral injections of 200 nL pAAV-hSyn-hChR2(H134R)-mCherry (Addgene #26976) were administered in the primary motor cortex (AP +1.5, ML ±1.8, DL -0.7) or somatosensory cortex (AP -1.5, ML ±3.0, DL -0.7) of WT and eIF4E-TG mice. For the Ca^2+^ imaging experiment, 250 nL pGP-AAV-*syn*-FLEX-jGCaMP7s-WPRE (Addgene #104491)^129^ was bilaterally injected in the striatum (AP +0.8, ML -1.8, DV -2.4). Post-operative carprofen and buprenorphine were administered i.p. twice a day for 24 h (5 mg/kg and 0.1 mg/kg, respectively). Mice were allowed to recover for at least three weeks to permit sufficient viral expression.

#### Electrophysiology

##### Sample preparation

Acute brain slices were prepared using either sucrose (in mM: 10 NaCl, 2.5 KCl, 25 NaHCO3, 1.25 NaH2PO4, 7 MgCl2, 0.5 CaCl2, 180 sucrose, 10 glucose) for mEPSC/mIPSC recordings and fast-scan cyclic voltammetry (FSCV), or NMDG-based cutting solutions^135^ (in mM: 92 NMDG, 30 NaHCO3, 2.5 KCl, 20 HEPES, 2 thiourea, 1.25 NaH2PO4, 3 Na-pyruvate, 5 ascorbic acid, 25 glucose, 0.5 CaCl2, 10 MgCl2) titrated with HCl to pH 7.4, for NMDA-mEPSC, optogenetic and plasticity recordings. The slices were then stored in standard ACSF (in mM: 125 NaCl, 2.5 KCl, 25 NaHCO3, 1.25 NaH2PO4, 1 MgCl2, 2 CaCl2, 10 glucose) or HEPES-based holding solution, respectively (in mM: 92 NaCl, 30 NaHCO3, 2.5 KCl, 20 HEPES, 2 thiourea, 1.25 NaH2PO4, 3 Na-pyruvate, 5 ascorbic acid, 25 glucose, 2.5 CaCl2, 2 MgCl2 titrated with NaOH to pH 7.4). Coronal sections (250 μm) containing the striatum were prepared using a Leica vibratome (VT 1200S). Sucrose-prepared slices were incubated at 32°C–34°C for 30 min in oxygenated ACSF, whereas NMDG-prepared slices were stored in NMDG solution for 10 min at 32°C–34°C before transferring to HEPES holding solution. Slices were transferred to a recording chamber, where they were continuously perfused with oxygenated ACSF at a rate of 3 mL/min.

##### Recording protocol

Recordings were obtained using a patch clamp electrophysiology rig fitted with SciCam pro camera (Scientifica, United Kingdom) equipped with a 40 ×0.8 NA water-immersion objective (LUMPlanFLN, Olympus, United States) and Dodt contrast tube optics. Cell/slice visualisation and light delivery were performed using a CoolLED PE-300 Ultra LED. Recordings were obtained with a Multiclamp 700B amplifier (Molecular Devices) and Axon Digidata 1550B digitizer (Molecular Devices, United States), using pCLAMP 11 software (Molecular Devices, United States). Data were low pass filtered at 2–10 kHz depending on protocol. For all recordings, borosilicate glass capillaries were used to prepare electrodes with 3–5 MΩ tip resistance. Recordings were not corrected for liquid junction potentials, and series resistance compensation was not applied during any of the recordings. For all recordings, series resistance was monitored and recordings were excluded if series resistance exceeded 30 MΩ or changed by over 20%, or in the event of declining cell health.

All recordings were done at 30°C–34°C. To detect miniature glutamate-mediated excitatory synaptic currents (mEPSCs), electrodes were filled with intracellular solution (IS) containing the following (in mM): 115 K-gluconate, 20 KCl, 20 HEPES, 1 MgCl2, 2 MgATP, 0.2 NaGTP, adjusted to pH 7.25 with KOH. To detect miniature GABA-mediated inhibitory synaptic currents (mIPSCs), 20 mM K-gluconate plus 115 mM KCl was substituted for 115 mM K-gluconate and 20 KCl using the previous IS. All other recordings were obtained using a cesium methanesulfonate-based internal solution containing (in mM): 126 CsMeSO3, 20 KCl, 4 MgATP, 0.3 Na2-GTP, 8 Na2-phosphocreatine, 1 EGTA, 10 HEPES and 0.1 CaCl2 at pH 7.25 and osmolarity 300 mOsm.

AMPA receptor-mediated mEPSCs were recorded in presence of bath-applied TTX (1 μM) to block action potential generation and picrotoxin (100 μM) to block GABAA-receptor mediated synaptic transmission. Recordings of mIPSCs were performed with bath application of TTX (1 μM), and DNQX (10 μM) and DL-AP5 (40 μM) were included to block glutamatergic currents. NMDA-receptor derived mEPSCs were recorded with bath application of TTX (300 nM), picrotoxin (50 μM) and DNQX (10 μM) in Mg^2+^-free ACSF. Optogenetic recordings, plasticity and 2-photon recordings were done in the presence of picrotoxin (50 μM). D-AP5 (10 μM), SCH39166 (1 μM) and sulpiride (2 μM) were added to the recording solution in a subset of plasticity recordings to block NMDA-receptors, D1 or D2 dopamine receptors, respectively. The concentrations used were selected based on previous electrophysiological studies demonstrating effective receptor antagonism in striatal neurons and corticostriatal plasticity paradigms.^102^^,^^107^^,^^136^^,^^137^^,^^138^ Chemicals were purchased from Fisher Scientific, Tocris and HelloBio.

Whole-cell voltage-clamp recordings were performed on striatal neurons identified by TdTomato or eGFP fluorescence. For all recordings, the access resistance was monitored throughout the recording by applying hyperpolarizing 10 mV voltage steps. Recordings were discarded if access resistance exceeded 30 MΩ or changed by over 20%. All recordings were performed in voltage-clamp configuration, with membrane potential clamped at −70 mV unless stated otherwise and were not corrected for liquid junction potential. Miniature excitatory and inhibitory post-synaptic currents (EPSC and IPSC) recordings were obtained in gap-free mode for five-10 min. Optogenetic recordings were obtained using either single or paired (delivered 100 ms apart) 1 ms pulses of blue light from a CoolLED PE-300 Ultra LED module through the 40× objective, at a rate of 0.033 Hz. Light intensity was set for each cell to provide an average EPSC of 200 pA. For NMDA: AMPA recordings, light stimulation was first given at −70 mV holding potential for several sweeps, before raising the holding potential to +40 mV and recording for several more sweeps.

Plasticity recordings were performed using paired (50 ms) electrical stimulation at a rate of 0.05 Hz. Baseline EPSC was established over 10 min, and stimulation intensity was set for each cell to provide an average EPSC of 250 pA. Cells exhibiting unstable baseline were excluded from recordings. After a stable baseline recording was obtained, plasticity induction stimulation was given in the form of high-frequency stimulation at 100 Hz for 1 s, repeated four times with 10-s intervals. Each 1-s bout of high-frequency stimulation (HFS) was paired with postsynaptic depolarisation of the holding potential to 0 mV. The stimulation was increased to twice the intensity used during baseline stimulation and reduced to the original level for recording the post-stimulation response.

##### Fast-scan cyclic voltammetry

Carbon fiber electrodes were generated as previously described^68^ and filled with 1 M KCl. Electrodes were calibrated on each day of recording by measuring the response to known concentrations of dopamine (0.5, 1 and 2 μM). Striatal slices were placed in the recording chamber and supplied with a constant flow of oxygenated (95% O2/5% CO2) ACSF at 3 mL/min, maintained at 30°C–34°C.

Recordings were obtained using the Chem-clamp 5MEG amplifier (Dagan corporation, Minnesota, USA), using the Demon Voltammetry suite software.^132^ A triangular voltage wave (−0.4V to +1.2V at 400V/s) was applied to the slice through the recording electrode at a frequency of 0.1 Hz. Slices were stimulated with a bipolar electrode placed 100–200 μm from the recording electrode, using an Iso-Flex stimulus isolator (A.M.P.I.). Single pulses (1 ms) were applied every 90 s to determine baseline dopamine release. As above, HFS was applied at a frequency of 100 Hz for 1 s, repeated four times with 10-s intervals. Slices were excluded if baseline or post-HFS dopamine currents were unstable or below detection threshold.

##### Two-photon GCaMP7s-based calcium imaging

Calcium imaging experiments were performed on a Scientifica Hyperscope multiphoton imaging system (Scientifica, Uckfield, UK) equipped with a 16× water-immersion objective (Zeiss, NA 0.8). Two-photon excitation of GCaMP7s was provided by a Chameleon Vision 1 femtosecond Ti-Sapphire laser (Coherent) tuned to 920 nm. Emitted fluorescence was collected through ET525/50m emission filters (Chroma Technologies) and detected by bi-alkali photomultiplier tubes (PMTs; R9880U, Hamamatsu). Laser power at the sample plane was controlled via a voltage-controlled Pockels cell and maintained below 21 mW to minimise phototoxicity and photobleaching. Images were acquired using a resonant scanner in ScanImage software (version 2021.0.0.3baa4b74be) at a frame rate of 5.3 Hz with a zoom factor of 15, resulting in 5.04 pixels per μm.

To evoke somatic depolarisation, we gained voltage control using whole-cell patch clamp recordings of neurons visualised using infrared Dodt gradient contrast imaging and an AxioCam ICm1 camera (Zeiss) controlled with ZEN 3.4 software. Neurons were impaled with patch pipettes and filled with cesium methanosulfonate-based internal solution (see electrophysiology > recording protocol). SeTau-647 (30 μM, Hölzel biotech) was included in the pipette to fluorescently mark the recorded neuron. Neurons were voltage-clamped at −70 mV and intermittent step commands (500 ms) depolarised the neuron to 0 mV. Step commands and membrane voltage recordings were performed with WinWCP (StratChlyde University, v5.8.3) controlling an Axopatch 200B amplifier (Molecular Devices) via a vDAQ data acquisition interface (National Instruments). During depolarisation, a trigger signal was sent to ScanImage (MATLAB-based software) to synchronise the onset of two-photon image acquisition with the electrophysiological stimulation.

##### Analysis

All analysis of patch-clamp data was performed using Clampfit or the open-access Python-based software Stimfit,^131^ whereas FSCV data analysis was performed using Demon Voltammetry suite software.^132^ Miniature EPSCs and IPSC recordings were analyzed using template scaling for event detection.^139^ Events were filtered using a post-analysis threshold of four times the standard deviation of the baseline of each detected event. Evoked responses were analyzed from averages of at least 10 sweeps. Paired pulse ratio was analyzed by expressing the amplitude of the second EPSC over the first EPSC. NMDA:AMPA ratio was analyzed by expressing the amplitude of the +40 mV EPSC, 50 ms after stimulation, divided by the −70 mV EPSC. In plasticity recordings, the post-stimulation EPSC (first EPSC from the paired response) was calculated as an average of the EPSCs at minutes 25–30 after the plasticity induction protocol, which was then expressed as a percentage change from the average baseline EPSC (100%) during 10 min prior to stimulation. If the EPSC changed from baseline by over 10% (increase or decrease) at 30 min post-stimulation, the cell was considered to express long-term plasticity.

FSCV recordings of HFS-evoked dopamine release were analyzed by expressing the size of the first peak elicited with HFS as a percentage change from the average baseline dopamine release (100%) elicited from five sweeps prior to HFS.

Two-photon GCaMP7s image analysis was performed using ImageJ software (v1.54p). Soma and dendrites were defined as regions of interest (ROIs), and mean fluorescence intensity values were extracted from each ROI across the time series. Background fluorescence was subtracted from each frame prior to further analysis. Relative changes in fluorescence (ΔF/F_0_) were calculated over time. Dendritic ROIs were selected at distances greater than 60 μm from the soma, with a maximum of five dendritic measurements per neuron.

#### Immunofluorescence

The process of Lucifer Yellow filling was followed as previously described.^69^^,^^140^ Mice were transcardially perfused with 4% paraformaldehyde (PFA) in 0.1 M PBS (pH 7.4), and brains were fixed for a further 4 h at 4 °C, before slicing at 200 μm using a Leica VT100s vibratome. Striatal cells were filled with 8% Lucifer Yellow CH lithium salt (Thermo Fisher Scientific; S32354) in PBS and Tris-HCl via iontophoretic current injection of 2–5 nA over 5–10 min. To amplify the fluorescent signal from biotin-conjugated Lucifer Yellow, slices were incubated for 48 h in Streptavidin Alexa Fluor-488 (Thermo Fisher Scientific, 1:200) in 0.6% Triton X-100 in TBS. Slices were incubated overnight in 2% NGS/0.6% TBS-triton with goat anti-mouse Alexa Fluor 488 Conjugate (Thermo Fisher Scientific, 1:500). Dendritic sections were imaged with a Zeiss LSM 800Airyscan microscope with a 63×/1.4 oil immersion objective (Art.Nr 420782-9900-799). Dendritic segments selected for analysis were located approximately 50 μm from the soma and were on average 22–24 μm in length. Dendritic spine density and subtype analysis was performed using the Imaris software 9.5.1 (Oxford Instruments). Dendritic spines subtypes were classified using the Imaris Spine Classification XTension (Oxford Instruments). Spine subtypes were classified automatically according to morphological criteria based on spine length, head width, and neck width. Stubby spines were defined as having a spine length ≤1 μm and a head width ≤ 2 × neck width; thin spines as having a spine length >1 μm and a head width ≤ 2 × neck width; and mushroom spines as having a head width > 2 × neck width. Classification parameters were adapted from previously established methods.^141^^,^^142^^,^^143^^,^^144^

To assess the specificity of channelrhodopsin expression in the optogenetic electrophysiological experiment, 30–40 μm coronal brain slices were prepared in PBS. Slices containing striatum were selected for immunostaining. Slices were permeabilised for 5 min in TBS containing 1:10 methanol and 1:3 hydrogen peroxide. A second permeabilisation step was performed using TBS with 0.2% Triton X-100 (TBS-Triton) and incubated for 15 min. Slices were incubated with primary antibody overnight at 4 °C (Rockland 600-401-379 rabbit anti-RFP; 1:1000) and then incubated with secondary antibody for 45–60 min at room temperature (Thermo Fisher Scientific A-11011 anti-rabbit 568; 1:500). Sections were imaged at 10× with a Zeiss LSM800 confocal microscope, and images were analyzed using the DMC-BrainMAP Napari plug-in.^81^

### Quantification and statistical analysis

All statistical analyses were performed using GraphPad Prism or R. N-values indicate number of mice and *n*-values indicate the number of cells, dendrites or slices. Data are presented as median ± quartiles or mean ± SEM. Data distribution was assessed for normality prior to parametric testing. Depending on data distribution and experimental design, two-tailed Student’s t-tests, Mann-Whitney U-tests, Chi-squared tests or linear mixed-effects models were used as appropriate.

Multiple neurons, dendrites, or slices were sampled from individual mice to account for biological and technical variability across preparations, consistent with standard practices in slice electrophysiology and imaging studies. To account for the nested structure of the data and non-independence of observations obtained from the same animal, datasets that met assumptions for parametric analysis were additionally analyzed using linear mixed-effects models (LMMs) fitted by restricted maximum likelihood (REML), with genotype as a fixed effect and mouse identity as a random effect. Assumptions were assessed by inspection of model residuals. For datasets that did not meet assumptions for parametric analyses, non-parametric tests were retained as indicated. Degrees of freedom were estimated using the Kenward–Roger method. Analyses were performed in R (version 4.6.0) using the lme4, lmerTest, and emmeans packages. The R script used for mixed-effects model analyses is provided in Data S1, and the corresponding data table arrangement is provided in Data S2.

In all cases, ∗*p* < 0.05, ∗∗*p* < 0.01, ∗∗∗*p* < 0.001 and ∗∗∗∗*p* < 0.0001; ns *p* > 0.05. Interaction effects were denoted as #*p* < 0.05, ##*p* < 0.01, ###*p* < 0.001 and ####*p* < 0.0001. Key statistical parameters, including N and *n* values, test statistics, *p*-values, and mixed-effects model outputs where applicable, are provided in Tables S1–S9.

## Supplemental information

Document S1. Figure S1 and Tables S1–S9

Data S1. R script used for linear mixed-effects model analysis

Data S2. Data table arrangement used for the analyses in Data S1

Data S3. Readme file describing the contents and use of Data S1 and Data S2
