# Supplementary material for: Altered striatal long-term potentiation in the eIF4E-TG mouse model of autism spectrum disorder
Source: iScience. 2026 Sep 3;29(9):117278. doi: 10.1016/j.isci.2026.117278 (PMC13571666; doi:10.1016/j.isci.2026.117278)
Supplement: Supplementary file 1 [file mmc1.pdf]

iScience, Volume *29*

Supplemental information

Altered striatal long-term potentiation in the eIF4E-TG mouse model of autism spectrum disorder

Alina Aaltonen, Jone Razquin, Daniel R. Garton, Julia Oyrer, Chiara Criscuolo, Ori J. Lieberman, Eric Klann, Anders Borgkvist, and Emanuela Santini

Supplementary figures

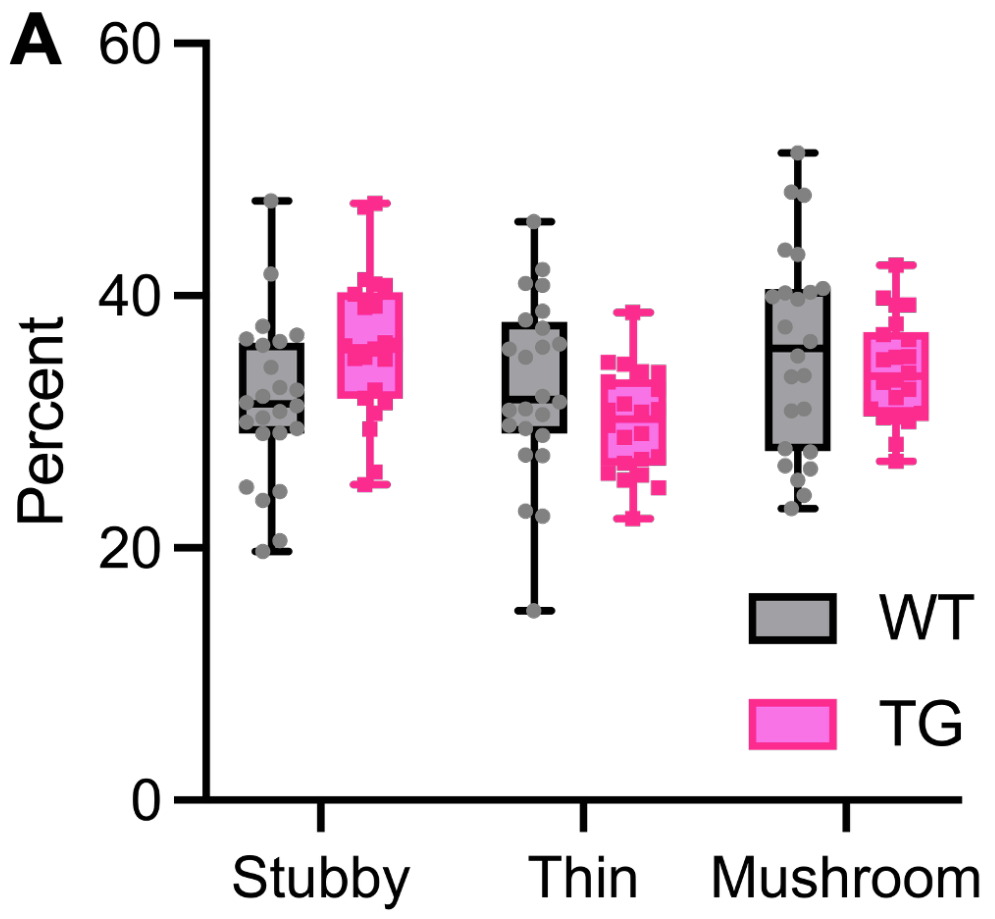

Figure S1. Dendritic spine subtype analysis, related to Figure 1

A) Percent of each type of spine (stubby, thin, and mushroom) identified from images analysed in Figure 1. Each dot represents a cell. To account for the nested structure of the data (multiple cells analysed per mouse), group differences in spine subtype percentage were independently assessed using a linear mixed-effects model (LMM) with group (WT vs TG) as a fixed effect and mouse identity as a random effect, fitted by restricted maximum likelihood (REML). Degrees of freedom were estimated using the Kenward-Roger method. Multiple comparisons were corrected for using P-values were corrected using the Benjamini-Hochberg false discovery rate (FDR). No significant differences were observed between the genotypes. For full details of statistical analysis, refer to Table S6.

Table S1 related to Figure 1. Summary statistics for dendritic spine density measurements in striatal SPNs from WT and eIF4E-TG mice.

	WT			TG				
Parameter	Mean +/- SEM	25%, median, 75%	N (mice) n (slices/dendrites)	Mean +/- SEM	25%, median, 75%	N (mice) n (slices/dendrites)	Test value and type	p-value
SPN spine density	1.931 +/- 0.073	1.661, 1.919, 2.159	3, 24	2.583 +/- 0.066	2.39, 2.59, 2.804	3, 22	LMM w/ mouse as random effect, kenward-roger method; t(3.96) = 5.80	** **0.0045**

Table S2 related to Figure 2. Summary statistics for spontaneous excitatory and inhibitory synaptic transmission in striatal SPNs.

	WT			TG				
Parameter	Mean +/- SEM	25%, median, 75%	N (mice) n (cells)	Mean +/- SEM	25%, median, 75%	N (mice) n (cells)	Test value and type	p-value
mEPSC frequency	1.025 +/- 0.117	0.722, 0.906, 1.356	7, 19	2.336 +/- 0.21	1.822, 2.390, 2.951	5, 16	LMM w/ mouse as random effect, kenward-roger method; t(7.00) = -5.41	*** **0.001**
mEPSC amplitude	14.32 +/- 0.953	10.38, 14.45, 18.33	7, 19	10.68 +/- 0.693	8.527, 10.81, 12.78	5, 16	LMM w/ mouse as random effect, kenward-roger method; t(8.30) = 2.37	* **0.0439**
mIPSC frequency	1.066 +/- 0.134	0.689, 0.99, 1.134	8, 17	1.044 +/- 0.102	0.742, 1.072, 1.165	5, 16	LMM w/ mouse as random effect, kenward-roger method; t(8.08) = 0.185	ns 0.8578
mIPSC amplitude	32.03 +/- 1.57	27.78, 32.6, 36.5	8, 17	21.45 +/- 1.692	15.39, 21.33, 26.38	5, 15	LMM w/ mouse as random effect, kenward-roger method; t(7.28) = 4.52	** **0.0025**
NMDA mEPSC frequency	3.434 +/- 0.438	1.89, 3.07, 5.00	5, 17	5.473 +/- 0.526	3.55, 6.15, 7.18	6, 15	LMM w/ mouse as random effect, kenward-roger method; t(6.79) = -2.85	* **0.0255**
NMDA mEPSC amplitude	8.838 +/- 0.418	7.475, 8.67, 9.97	5, 17	9.883 +/- 0.59	8.81, 10.41, 10.52	6, 15	LMM w/ mouse as random effect, kenward-roger method; t(6.79) = -1.40	ns 0.2063

Table S3 related to Figure 3. Summary statistics for baseline excitatory synaptic transmission at corticostriatal and thalamostriatal synapses.

	WT			TG				
Parameter	Mean +/- SEM	25%, median, 75%	N (mice) n (cells)	Mean +/- SEM	25%, median, 75%	N (mice) n (cells)	Test value and type	p-value
M1 amplitude	609.1 +/- 110.4	281, 374.5, 954.1	4, 21	496.2 +/- 134.8	45.24, 219.6, 638.9	7, 27	LMM w/ mouse as random effect, kenward-roger method; t(9.77) = 0.141	ns 0.8904
M1 PPR	0.5658 +/- 0.043	0.4366, 0.5612, 0.7063	4, 21	0.5288 +/- 0.04	0.3552, 0.5542, 0.7295	7, 32	LMM w/ mouse as random effect, kenward-roger method; t(9.71) = 0.370	ns 0.7195
M1 NMDA: AMPA	0.3146 +/- 0.021	0.2275, 0.3078, 0.3705	4, 20	0.2813 +/- 0.021	0.2129, 0.2616, 0.3283	7, 21	LMM w/ mouse as random effect, kenward-roger method; t(7.88) = 0.583	ns 0.5763
S1 amplitude	318.8 +/- 63.06	73.72, 194, 534.9	9, 26	411.5 +/- 74.72	125.2, 270.1, 565.6	7, 28	LMM w/ mouse as random effect, kenward-roger method; t(10.7) = -0.981	ns 0.3791
S1 PPR	0.5227 +/- 0.049	0.3263, 0.4868, 0.6681	9, 26	0.5727 +/- 0.038	0.4289, 0.5616, 0.6985	7, 28	LMM w/ mouse as random effect, kenward-roger method; t(10.7) = -0.792	ns 0.4459
S1 NMDA: AMPA	0.2573 +/- 0.027	0.179, 0.25, 0.34	9, 25	0.2076 +/- 0.019	0.133, 0.198, 0.274	7, 26	LMM w/ mouse as random effect, kenward-roger method; t(10.4) = 1.455	ns 0.1751

Table S4 related to Figure 4. Summary statistics for long-term potentiation and dopamine release experiments.

	WT			TG				
Parameter	Mean +/- SEM	25%, median, 75%	N (mice) n (cells)	Mean +/- SEM	25%, median, 75%	N (mice) n (cells)	Test value and type	p-value
Plasticity distribution							X^2=4.413, Chi-square test	* **0.0418**
EPSC change (LTP)	137.5 +/- 7.328	116.9, 135.7, 145.7	9, 11	235.1 +/- 28.79	148.9, 208.7, 276.3	9, 12	LMM w/ mouse as random effect, kenward-roger method; t(15.9) = -2.951	** **0.0094**
EPSC change, ACSF	107.395 +/- 9.017	83.713, 111.55, 135.77	17, 21	212.986 +/- 28.82	146.61, 189.67, 269.43	10, 14	LMM, mouse as random effect, Kenward-Roger method, Tukey corrected:<br><br>Genotypeeffect: F(1, 36.5) = 15.19,<br><br>ACSF: t(47.4) = 4.34<br>SCH/Sulpiride: t(28.4) = 2.54<br><br>AP5<br>(t(52.4) = 0.46)	Genotype: ***** p=0.0004;**<br><br>**ACSF: **** p<0.0001**<br><br>AP5: n.s. p = 0.64<br><br>**SCH/Sulpiride: * p=0.017**
EPSC change, AP5	124.077 +/- 13.03	96.044, 106.67, 160.61	7, 9	136.405 +/- 17.97	90.261, 128.28, 171.86	8, 11		
EPSC change, SCH/sulpiride	99.656 +/- 2.917	95.229, 97.067, 105.553	4, 8	194.129 +/- 16.032	154.613, 207.93, 227.033	4, 8		
Baseline DA release	0.6498 +/- 0.0489	0.4489, 0.5279, 1.014	6, 18	0.3782 +/- 0.03745	0.2533, 0.3604, 0.4347	6, 21	LMM w/ mouse as random effect, Kenward-Roger method; t(9.81) = -3,321	LMM * * **p= 0.0079**
HFS DA release %	68.07 +/- 14.08	28.05, 55.25, 109.4	4, 14	60.63 +/- 9.024	39.3, 60.71, 90.16	4, 17	LMM w/ mouse as random effect, Kenward-Roger method; t(4.76) = 0.469	ns 0.6595

Table S5 related to Figure 5. Summary statistics for dendrite-to-soma Ca^{2+} fluorescence measurements in striatal SPNs.

	WT			TG				
Parameter	Mean +/- SEM	25%, median, 75%	N (mice) n (cells)	Mean +/- SEM	25%, median, 75%	N (mice) n (cells)	Test value and type	p-value
Dendritic: somatic GCaMP7s peak fluorescence	1.302 +/- 0.2696	0.2616, 0.7021, 2.178	4, 30	2.274 +/- 0.2664	0.6892, 1.858, 3.166	5, 47	u=422, Mann-Whitney	** **0.0051**

Table S6 related to Figure S1. Summary statistics for dendritic spine subtype analysis in striatal SPNs.

	WT			TG				
Spine subtype	Mean +/- SEM	25%, median, 75%	N (mice) n (cells)	Mean +/- SEM	25%, median, 75%	N (mice) n (cells)	Test value and type	p-value
Stubby	31.633 +/- 1.304	29.074, 31.401, 36.286	3, 24	36.001 +/- 1.235	31.808, 35.885, 40.297	3, 22	LMM w/ mouse as random effect, kenward-roger method; t(3.95) = -1.766	0.1530, FDR adjusted: 0.2295
Thin	32.767 +/- 1.435	29.074, 31.789, 37.916	3, 24	29.927 +/- 0.860	26.540, 30.240, 33.376	3, 22	LMM w/ mouse as random effect, kenward-roger method; t(3.99) = 1.820	0.143, FDR adjusted: 0.2295
Mushroom	35.601 +/- 1.662	27.681, 35.803, 40.520	3, 24	34.072 +/- 0.868	30.590, 33.613, 37.127	3, 22	LMM w/ mouse as random effect, kenward-roger method; t(3.95) = 0.358	0.739, FDR adjusted: 0.7390

Table S7 of cell-type analysis related to Figure 1, 2 and 4. Two-way ANOVA results for genotype and SPN subtype comparisons.

	WT		TG			
	D1-SPNs	D2-SPNs	D1-SPNs	D2-SPNs		
Parameter	Mean +/- SEM, N/n		Mean +/- SEM, N/n		Test value and type	p-value
Spine density	2.152 +/- 0.075<br><br>N=3, n=35	1.758 +/- 0.073<br><br>N=3, n=39	2.684 +/- 0.059<br><br>N=3, n=33	2.482 +/- 0.077<br><br>N=3, n=36	Two-way-ANOVA Genotype: $F_{(1, 139)}$=75.85; Cell type: $F_{(1, 139)}$ =17.09; Genotype x Cell type: $F_{(1, 139)}$= 1.766	Genotype: ****** <0.0001;** Cell type: **** **<0.0001**; Genotype x Cell type: ns 0.1860
mEPSC frequency	1.085 +/- 0.203<br><br>N=7, n=10	0.958 +/- 0.114<br><br>N=7, n=9	2.272 +/- 0.323<br><br>N=5, n=7	2.386 +/- 0.294<br><br>N=5, n=9	Two-way-ANOVA Genotype: $F_{(1, 31)}$=29.93; Cell type: $F_{(1, 31)}$ =0.0007; Genotype x Cell type: $F_{(1, 31)}$= 0.2548	Genotype: ****** <0.0001;** Cell type: ns 0.9798; Genotype x Cell type: ns 0.6173
mEPSC amplitude	-13.319 +/- 1.302<br><br>N=7, n=10	-15.427 +/- 1.378<br><br>N=7, n=9	-11.041 +/- 1.215<br><br>N=5, n=7	-10.398 +/- 0.848<br><br>N=5, n=9	Two-way-ANOVA Genotype: $F_{(1, 31)}$=8.829; Cell type: $F_{(1, 31)}$ =0.3546; Genotype x Cell type: $F_{(1, 31)}$= 1.252	Genotype: **** 0.0057;** Cell type: ns 0.5558; Genotype x Cell type: ns 0.2717
mIPSC frequency	0.792 +/- 0.086<br><br>N=7, n=7	1.259 +/- 0.202<br><br>N=8, n=10	1.041 +/- 0.072<br><br>N=8, n=8	1.048 +/- 0.198<br><br>N=8, n=8	Two-way-ANOVA Genotype: $F_{(1, 29)}$=0.0136; Cell type: $F_{(1, 29)}$ =2.047; Genotype x Cell type: $F_{(1, 29)}$= 1.927	Genotype: ns 0.9080; Cell type: ns 0.1632; Genotype x Cell type: ns 0.1757
mIPSC amplitude	-35.067 +/- 1.962<br><br>N=7, n=7	-29.905 +/- 2.105<br><br>N=8, n=10	-19.658 +/- 2.575<br><br>N=8, n=8	-23.503 +/- 2.041<br><br>N=8, n=8	Two-way-ANOVA Genotype: $F_{(1, 29)}$=23.57; Cell type: $F_{(1, 29)}$ =0.0859; Genotype x Cell type: $F_{(1, 29)}$= 4.018	Genotype: ****** <0.0001;** Cell type: ns 0.7717; Genotype x Cell type: ns 0.0548
NMDA mEPSC frequency	3.908 +/- 0.603<br><br>N=5, n=9	2.901 +/- 0.623<br><br>N=5, n=8	5.249 +/- 0.787<br><br>N=6, n=8	5.729 +/- 0.735<br><br>N=6, n=7	Two-way-ANOVA Genotype: $F_{(1, 28)}$=9.173; Cell type: $F_{(1, 28)}$ =0.1465; Genotype x Cell type: $F_{(1, 28)}$= 1.166	Genotype: **** 0.0052;** Cell type: ns 0.7048; Genotype x Cell type: ns 0.2894
NMDA mEPSC amplitude	8.892 +/- 0.6<br><br>N=5, n=9	8.676 +/- 0.615<br><br>N=5, n=8	10.436 +/- 0.987<br><br>N=6, n=8	9.25 +/- 0.562<br><br>N=6, n=7	Two-way-ANOVA Genotype: $F_{(1, 28)}$=1.987; Cell type: $F_{(1, 28)}$ =1.076; Genotype x Cell type: $F_{(1, 28)}$= 0.3744	Genotype: ns 0.1697; Cell type: ns 0.3085; Genotype x Cell type: ns 0.5455
EPSC change (LTP)	135.881 +/- 8.669<br><br>N=5, n=5	138.832 +/- 12.082<br><br>N=6, n=6	198.386 +/- 25.203<br><br>N=5, n=5	261.33 +/- 45.024<br><br>N=7, n=7	Two-way-ANOVA Genotype: $F_{(1, 19)}$=8.843; Cell type: $F_{(1, 19)}$ =1.123; Genotype x Cell type: $F_{(1, 19)}$= 0.927	Genotype: **** 0.0078;** Cell type: ns 0.3025; Genotype x Cell type: ns 0.3477

Table S8 of sex analysis related to Figure 2 and 4. Two-way ANOVA results for genotype and sex comparisons.

	WT		TG			
	Male	Female	Male	Female		
Parameter	Mean +/- SEM, N/n		Mean +/- SEM, N/n		Test value and type	p-value
mEPSC frequency	1.057 +/- 0.16<br>N=4, n=13	0.954 +/- 0.146<br>N=3, n=6	2.828 +/- 0.249<br>N=3, n=6	2.041 +/- 0.269<br>N=2, n=10	Two-way-ANOVA Genotype: $F_{(1, 31)}$=38.18; Sex: $F_{(1, 31)}$ =3.706; Genotype x Sex: $F_{(1, 31)}$= 2.178	Genotype: **** **<0.0001;** Sex: ns 0.0634; Genotype x Sex: ns 0.1501
mEPSC amplitude	-14.493 +/- 1.122<br>N=4, n=13	-13.936 +/- 1.939<br>N=3, n=6	-10.138 +/- 1.474<br>N=3, n=6	-11.004 +/- 0.725<br>N=2, n=10	Two-way-ANOVA Genotype: $F_{(1, 31)}$=7.652; Sex: $F_{(1, 31)}$ =0.0138; Genotype x Sex: $F_{(1, 31)}$= 0.2919	Genotype: ** **0.0095;** Sex: ns 0.9072; Genotype x Sex: ns 0.5929
mIPSC frequency	1.224 +/- 0.248<br>N=4, n=7	0.956 +/- 0.149<br>N=4, n=10	1.019 +/- 0.193<br>N=3, n=8	1.069 +/- 0.083<br>N=2, n=8	Two-way-ANOVA Genotype: $F_{(1, 29)}$=0.0694; Sex: $F_{(1, 29)}$ =0.3984; Genotype x Sex: $F_{(1, 29)}$= 0.8451	Genotype: ns 0.7942; Sex: ns 0.5329; Genotype x Sex: ns 0.3655
mIPSC amplitude	-33.786 +/- 2.029<br>N=4, n=7	-30.802 +/- 2.262<br>N=4, n=10	-20.838 +/- 1.585<br>N=3, n=8	-22.154 +/- 3.289<br>N=2, n=8	Two-way-ANOVA Genotype: $F_{(1, 29)}$=20.81; Sex: $F_{(1, 29)}$ =0.1243; Genotype x Sex: $F_{(1, 29)}$= 0.8253	Genotype: **** **<0.0001;** Sex: ns 0.727; Genotype x Sex: ns 0.3714
EPSC change (LTP)	140.147 +/- 9.675<br>N=7, n=8	130.29 +/- 8.691<br>N=2, n=3	243.322 +/- 48.561<br>N=5, n=6	226.884 +/- 35.517<br>N=4, n=6	Two-way-ANOVA Genotype: $F_{(1, 19)}$=8.381; Sex: $F_{(1, 19)}$ =0.1452; Genotype x Sex: $F_{(1, 19)}$= 0.0091	Genotype: ** **0.0093;** Sex: ns 0.7074; Genotype x Sex: ns 0.925

Table S9 for membrane properties related to Figure 4 and 2. Summary statistic for passive membrane properties of striatal SPNs recorded during electrophysiological experiments.

**LTP recordings**	**WT**		**TG**			
Parameter	Mean +/- SEM	N (mice) n (cells)	Mean +/- SEM	N (mice) n (cells)	Test value and type	p-value
Capacitance (pF)	98.52 +/- 4.825	32, 38	96.72 +/- 5.894	26, 33	t=0.2391, unpaired t-test	ns 0.8117
Resistance (MΩ)	205.9 +/- 15.57	32, 38	171.4 +/- 11.62	26, 33	t=1.731, unpaired t-test	ns 0.0879
Time constant (ms)	1.347 +/- 0.058	32, 38	1.44 +/- 0.088	26, 33	t=0.8964, unpaired t-test	ns 0.3732
Holding current (pA)	-67.98 +/- 6.107	32, 38	-59.60 +/- 5.866	26, 33	t=0.983, unpaired t-test	ns 0.3291
Access resistance (Ra): baseline	17.762 +/- 0.803	32, 38	19.711 +/- 1.033	26, 33	Two-way RM-ANOVA Genotype: $F_{(1, 69)}$=1.598; Time: $F_{(1, 69)}$ =3.888; Genotype x Time: $F_{(1, 69)}$= 1.716	Genotype: ns 0.2105; Time: ns 0.0527; Genotype x Time: ns 0.1947
Ra: post-stimulation	18.753 +/- 0.807	32, 38	19.911 +/- 0.952	26, 33		
**NMDA mEPSC***	**WT**		**TG**			
Parameter	Mean +/- SEM	N (mice) n (cells)	Mean +/- SEM	N (mice) n (cells)	Test value and type	p-value
Capacitance (pF)	83.49 +/- 5.839	5, 17	89.95 +/- 7.773	6, 15	t=0.6736, unpaired t-test	ns 0.5057
Resistance (MΩ)	166 +/- 22.1	5, 17	141.1 +/- 24.98	6, 15	t=0.7488, unpaired t-test	ns 0.4598
Time constant (ms)	1.571 +/- 0.117	5, 17	1.466 +/- 0.104	6, 15	t=0.6642, unpaired t-test	ns 0.5116
Holding current (pA)	-137.6 +/- 13.09	5, 17	-123.1 +/- 11.15	6, 15	t=0.8334, unpaired t-test	ns 0.4112

NMDA mEPSC*= NMDA-mEPSC recordings were performed in Mg2+-free ACSF in the presence of DNQX and picrotoxin.
